# Initial Exploration of the In Vitro Activation of GLP-1 and GIP Receptors and Pancreatic Islet Cell Protection by Salmon-Derived Bioactive Peptides

**DOI:** 10.3390/md22110490

**Published:** 2024-10-30

**Authors:** Crawford Currie, Christian Bjerknes, Bomi Framroze

**Affiliations:** 1 HBC Immunology Inc., Menlo Park, CA 2043, USA; bf@hbcimmunology.com; 2 Hofseth BioCare, 6003 Ålesund, Norway; chbj@hofsethbiocare.no

**Keywords:** weight management, metabolic health, healthy ageing, GLP-1 receptor, GIP receptor, ALOX12, pancreatic islet cells, soluble protein hydrolysate, salmon protein hydrolysate, bioactive peptides

## Abstract

This study examines the in vitro effects of a soluble protein hydrolysate (SPH) derived from Atlantic salmon (Salmo salar) on incretin receptor activity and pancreatic islet cell protection to explore the mechanisms underlying SPH’s observed benefits on weight loss and metabolic health in overweight individuals. SPH demonstrated a dose-dependent enhancement of glucagon-like peptide-1 (GLP-1) and gastric inhibitory polypeptide (GIP) receptor activity, with significant increases of 2.4-fold (*p* < 0.05) and 2.6-fold (*p* < 0.01) at 10 mg/mL, respectively, compared to the control. Pancreatic islet cell assays showed a substantial proliferation effect, with up to a 57% increase at 50 µL/well, indicating potential protective properties against inflammation-induced cell loss. Notably, the smallest SPH peptide fraction (<1000 Da) exhibited GLP-1 agonist activity comparable to semaglutide, a widely used therapeutic agent, underscoring SPH’s potential efficacy in modulating metabolic pathways. These results suggest that SPH not only enhances key incretin signaling but also promotes islet cell health, positioning it as a promising dietary intervention to improve age-related metabolic health, including the weight gain and underlying adverse metabolic changes frequently encountered through the menopause.

## 1. Introduction

Diet, exercise and sleep are central to the maintenance of good health [1]. However, consistently attaining these three pillars of health is difficult in the context of modern lifestyles. Time pressures and financial constraints can necessitate shortcuts in terms of dietary choices, such as the selection of calory-dense (and highly palatable) processed foods. This, coupled with limited opportunities for physical activity, has contributed to a significant increase in obesity rates over the past few decades, with 38% of the world’s population now classified as overweight or obese [2]. 

Ultimately, the health consequences of excess weight can be devastating, with significantly increased rates of diabetes, cardiovascular disease and cancer, all a consequence of chronic inflammation and oxidative stress in the body. Tackling obesity ultimately will require preventative strategies, including targeting the progressive weight gain that commonly occurs with ageing [3,4]. Ageing is associated with a gradual increase in inflammation and oxidative stress within the body. This multifactorial process is influenced by factors such as a sedentary lifestyle, weight gain and an accumulation of senescent cells [5]. This not only places the individual at an increased risk of conditions such as diabetes, but also an accelerated loss of lean body mass and the development of frailty (sarcopenia) [6,7]. With an ageing population, strategies for weight management become ever more urgent to help improve overall health and quality of life [1,8]. 

Until quite recently, weight management strategies were limited to reducing calorie intake and increasing levels of exercise. However, long-term adherence to such approaches is challenging and ultimately the biological drive to eat is very difficult to oppose chronically [9]. Frustratingly, pharmacologic approaches were either ineffective or associated with unacceptable risk [10,11]. Fortunately, the emergence of glucagon-like peptide-1 (GLP-1)-based therapies has transformed weight management. These therapies enable obese subjects to frequently lose 15% or more of their body weight and have demonstrated major health benefits, including a reduced risk of heart attack, stroke and renal dysfunction [12]. However, for individuals who are overweight and otherwise healthy, interventions to support effective weight management remain sparse. While at first glance this may not appear to be a significant unmet need, it should be considered that the only way to fully tackle the obesity crisis will be through the prevention of overweight individuals developing obesity.

Marine proteins are well recognized as a source of bioactive peptides (BPs) [13]. These are typically small peptide fragments, ranging from three to twenty amino acids in length, and act as biological regulators that enhance health. The reported health benefits of marine BPs are diverse, and therefore, individual marine protein hydrolysates can display an array of bioactivities [14]. These include anti-hypertensive, antioxidant and glucoregulatory actions [15]. Whilst inhibition of dipeptidyl peptidase IV (DPP-IV) has been demonstrated with several marine-derived BPs, to date, direct GLP-1 agonism has not been. DPP-IV inhibition slows the breakdown of GLP-1; however, this is a relatively weak mode of action. GLP-1 agonists show considerably greater improvements in the regulation of blood glucose levels and enable significant weight loss. In contrast, DPP-IV inhibitors provide only moderate reductions in blood glucose and are merely weight neutral [16]. 

SPH, a soluble protein hydrolysate, contains a mixture of bioactive peptides derived through enzymatic hydrolysis of Norwegian Atlantic salmon (*Salmo salar*). Clinical and preclinical studies with SPH have consistently shown antioxidant and anti-inflammatory effects, improved metabolic profiles and enhanced levels of energy and vitality [17,18,19]. These benefits stem from the upregulation of antioxidant protective gene systems, including *FTH1* (ferritin heavy chain-1) and *HMOX1* (hemoxygenase-1), as well as the downregulation of the pro-inflammatory *ALOX12* (12-lipoxygenase) gene system by SPH. The downstream health effects include optimized levels of ferritin and hemoglobin and a 6%-to-7% reduction in body weight in overweight individuals [19,20]. In addition to reductions in body mass, increases in serum bile acid (+63%), adiponectin (+11%) and lipoprotein lipase (+15%) have also been observed, along with reductions in fasting plasma glucose (FPG) (−6%) and the pro-inflammatory cytokine IL-6 (−15%) [19,20]. 

This profile of SPH, characterized by weight reduction, lowered FPG and increased adiponectin levels, suggests potential GLP-1 agonist activity [19,21]. Furthermore, the peptides have demonstrated gut health benefits with an upregulation in *HMOX1* (hemoxygenase-1) expression and an increase in GLP-2 (glucagon-like peptide 2) receptor activity [22,23]. 

This study represents the first stage of research to investigate whether the SPH peptides activate GLP-1 and GIP (glucose-dependent insulinotropic polypeptide) receptors in vitro. Activation of these receptors is not only a well-established means of modulating nutrient intake, with associated weight loss, but is also likely to provide broad ranging health benefits [24,25]. The study also evaluated whether the bioactive peptides might support islet cell health, a crucial element of metabolic health via insulin production and secretion. Elevated levels of inflammatory mediators, including arachidonic acid, associated with *ALOX12* expression, have been implicated in the inflammatory milieu in obesity and islet cell death. Therefore, the downregulation of this gene system by SPH could feasibly provide a protective effect on pancreatic islet cells, a potentially important attribute in the context of an ageing and weight-challenged global population [26,27]. More broadly, utilizing marine-derived bioactives with the aim of maintaining better health and delaying the need for medical intervention could have broad ranging benefits for society. 

## 2. Results

### 2.1. GLP-1 Receptor Agonism Assay Results for SPH

**Figure 1:**
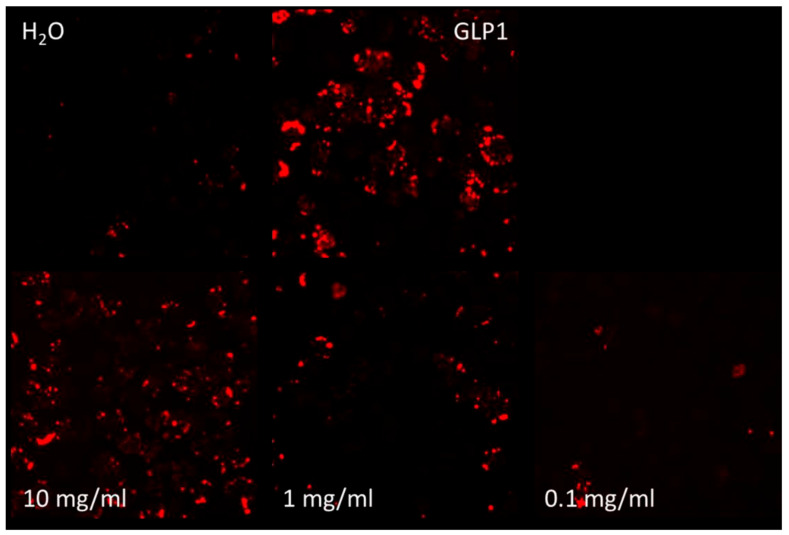
Images acquired with the Cell Insight High-Content Bioimager CX7 (ThermoFisher) show red cAMP-Nomad fluorescence intensity, indicating the level of GLP-1 receptor activation. Images compare exposure to water (H_2_O), a laboratory standard GLP-1 receptor agonist and three concentrations of SPH (all shown in the bottom row). The top right quadrant is intentionally left blank.

**Figure 2:**
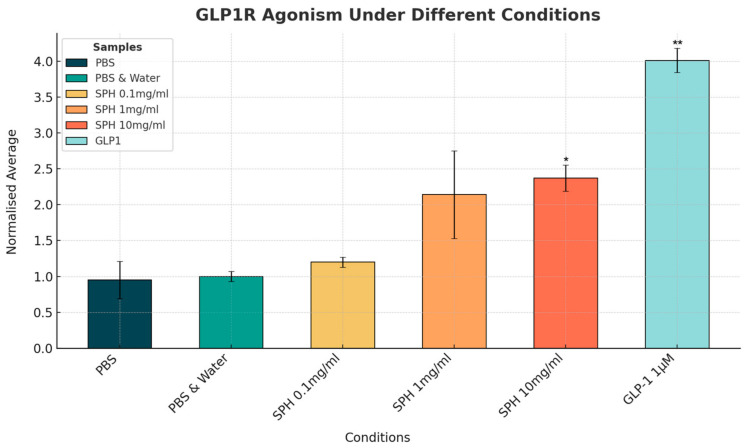
Normalized mean GLP-1 receptor activity measured by change in intracellular cyclic adenosine monophosphate (cAMP) levels. Results are expressed as fluorescence intensity (arbitrary units (a.u.)) of the red cAMP-Nomad biosensor. Each of the six conditions was analyzed in three independent experiments (three replicates per experimental group, n = 3), making a total of eighteen assessments. Data points represent the mean ± standard deviation for each test condition. The GLP-1 agonist dose of 1µM is equivalent to 0.004 mg/mL. * Indicates a *p*-value of >0.05, and ** indicates a *p*-value of <0.01 compared to PBS control.

The change in GLP-1 activity with SPH and with a GLP-1 agonist could be seen clearly on fluorescence intensity images (see Figure 1). SPH demonstrated a dose-dependent response in GLP-1 receptor activity, with the lowest concentration of 0.1 mg/mL showing minimal activity. In contrast, the 1 mg/mL and 10 mg/mL doses resulted in a 2-fold and 2.4-fold increase in GLP-1 receptor activation, respectively, compared to the negative control of phosphate-buffered saline (PBS) and PBS with water. The 10 mg/mL dose of SPH resulted in a significant increase in GLP-1 receptor activity (*p* < 0.05), and the 1 mg/mL showed a near-significant trend (*p* = 0.052). The GLP-1 receptor agonist (positive control) showed a significant 4-fold increase in GLP-1 receptor activity compared to the negative control (*p* < 0.01) at a concentration of 1 µM (see Figure 2). 

### 2.2. GLP-1 Agonist Activity in Fractionated SPH

**Figure 3:**
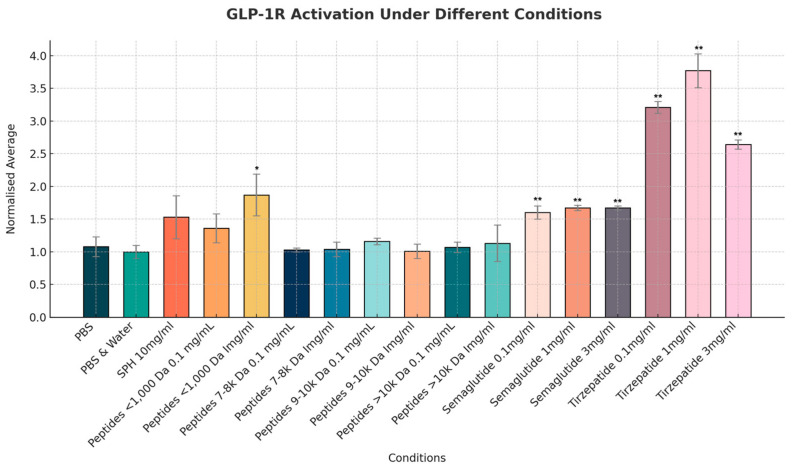
Normalized mean level of GLP-1 activity as measured by change in intracellular cAMP levels. Results are presented as the fluorescence intensity (arbitrary units/a.u.) of the red cAMP-Nomad biosensor. Each of the seventeen conditions was analyzed in three independent experiments (three replicates per experimental group, n = 3), making a total of fifty-one assessments. Data points represent the mean ± standard deviation for each test condition. * Indicates a *p*-value of >0.05, and ** indicates a *p*-value of <0.01 compared to PBS control.

Amongst the different fractions of SPH, a significant GLP-1 agonist effect was seen in the smallest peptide fraction consisting of peptides of less than 1000 Daltons (see Figure 3). These peptides, at a dilution of 1 mg/mL, showed a normalized mean increase of 1.87-fold in GLP-1 receptor activity which reached statistical significance versus the PBS control (*p* < 0.05), compared to a mean increase of 1.53-fold with SPH dosed at 10 mg/mL (which did not quite reach significance versus control, *p* = 0.058). The small peptide fraction also showed a similar agonist activity to the GLP-1 analogue semaglutide (Ozempic/Wegovy); however, the latter was at a higher significance level versus the control (*p* < 0.01). Tirzepatide (the dual GLP-1, GIP analogue, Mounjaro/Zepbound) showed an almost doubling in GLP-1 receptor activation compared to the small peptides and semaglutide. 

### 2.3. GIP Receptor Agonism Assay Results for SPH

**Figure 4:**
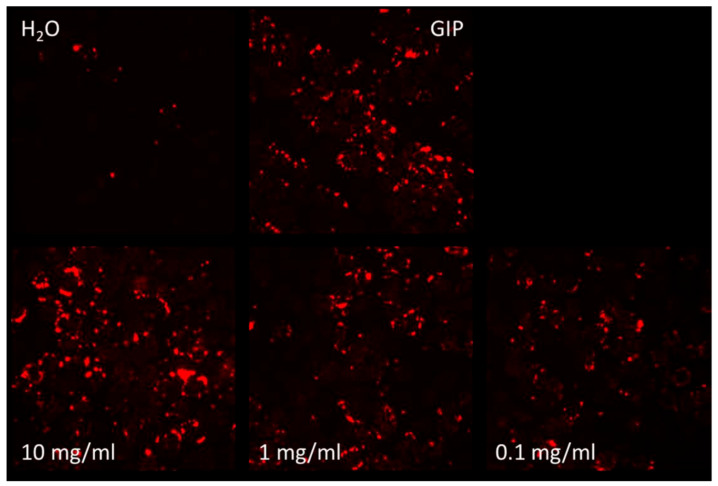
Images acquired with the Cell Insight High-Content Bioimager CX7 show red cAMP-Nomad fluorescence intensity illustrating GIP receptor activation levels. Images compare exposure to water (H_2_O), a laboratory standard GIP receptor agonist and three concentrations of SPH (all shown in the bottom row). The top right quadrant is intentionally left blank.

**Figure 5:**
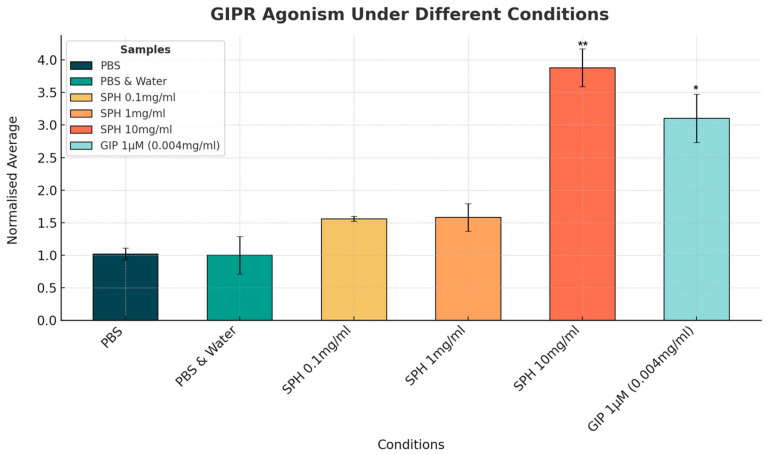
Normalized mean level of GIP receptor activity measured by changes in intracellular cAMP levels. Results are expressed as the fluorescence intensity (arbitrary units/a.u.) of the red cAMP-Nomad biosensor. Each of the six conditions was analyzed in three independent experiments (three replicates per experimental group, n = 3), making a total of eighteen assessments. Data points represent the mean ± standard deviation for each test condition. GIP agonist dose of 1 µM is equivalent to 0.004 mg/mL. * Indicates a *p*-value of <0.05, and ** indicates a *p*-value of <0.01 compared to PBS negative control.

The change in GIP activity with SPH and the GIP receptor agonist could be seen clearly on fluorescence imaging (see Figure 4). SPH demonstrated a dose-dependent response in GIP receptor activity. However, only the highest concentration of 10 mg/mL showed a clear upregulation in activity with a 2.6-fold increase in GIP receptor activation compared to the negative control (*p* < 0.01). The GIP receptor agonist (positive control), at a concentration of 1 µM, produced a 4-fold increase in GLP-1 receptor activation compared to PBS negative control (see Figure 5).

### 2.4. GIP Agonist Activity in Fractionated SPH

**Figure 6:**
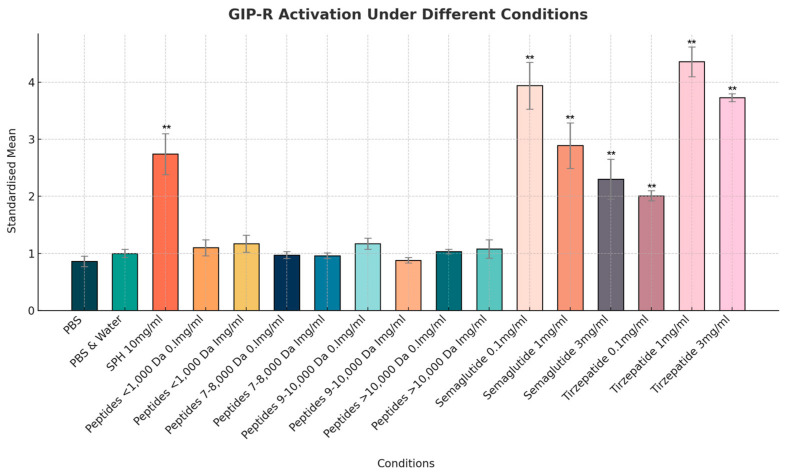
Normalized mean level of GIP activity as measured by change in intracellular cAMP levels. Results are expressed as the fluorescence intensity (arbitrary units/a.u.) of the red cAMP-Nomad biosensor. Each of the seventeen conditions was analyzed in three independent experiments (three replicates per experimental group, n = 3), making a total of fifty-one assessments. Data points represent the mean ± standard deviation for each test article. ** Indicates a *p*-value of <0.01 compared to PBS negative control.

While SPH at 10 mg/mL exhibited marked GIP agonist activity, with a normalized mean increase in GIP receptor activity of 2.8-fold (*p* < 0.01), none of the individual peptide fractions at 1 mg/mL elicited similar levels of activity, and all changes were non-significant (*p* > 0.05). This suggests that the observed activity in SPH is mediated by a peptide fraction between >1000 Da and <7000 Da. In comparison, semaglutide showed more than twice the level of GIP receptor activation, with a normalized mean level of 2.89 (a.u.), with 1 mg/mL compared to the maximum level of 1.17 (a.u.) seen with SPH peptides of <1000 Da and of 9000–10,000 Da. The activity of tirzepatide was again the higher with a 4.36-fold increase in GIP receptor activation (see Figure 6). 

### 2.5. Liquid Chromatography–Mass Spectrometry (LC-MS) Peptidomic Analysis

MS-based peptidomic analysis was undertaken to start the process of better characterizing the peptides contained within the <1000 Da dialysate fraction with GLP-1R agonist activity. This identified 1674 separate peptides within this fraction with a higher average size of 1490 Da (ranging from 724 Da to 2489 Da) than indicated by the dialysis methodology. Of the 1674 individual peptides, the Peptide Ranker on-line predictive tool identified that eighty of the peptides had a high likelihood of bioactivity, based on their peptide primary structure. This analysis was based on a rank threshold of 0.8, with an associated 6% false positive rate, as determined by Bioware [28].

### 2.6. Islet Cell Proliferation Assays

**Figure 7:**
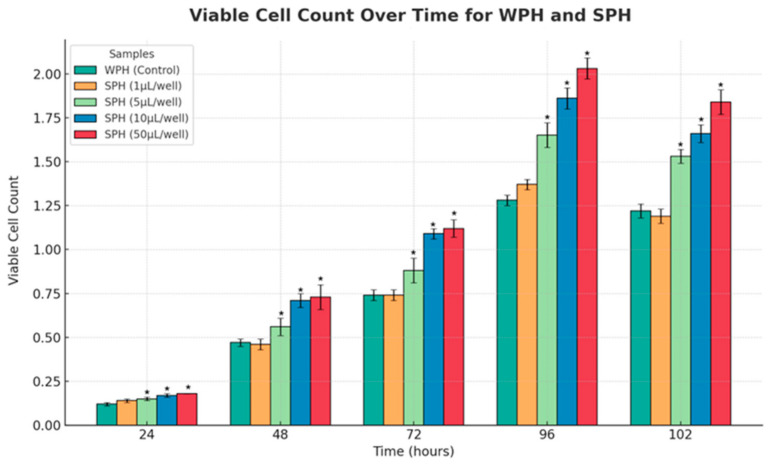
Effect of increasing doses of SPH peptides on the proliferation of pancreatic islet cells (ABC-TC4286 hPIC cells) from 24 h to 102 h, compared to control (WPH/whey protein hydrolysate at 10 µL/well). The concentration of WPH and SPH used is 1 mg/mL. Each of the five conditions was analyzed in three independent experiments at five different time points, making a total of seventy-five assessments. Data points represent the mean ± standard error of the mean for each test article. * Indicates a *p*-value of <0.01 compared to control (whey protein hydrolysate).

Using ABC-TC4286 hPIC cells, the potential of SPH peptides to enhance islet cell proliferation was compared to the whey protein hydrolysate (WPH) control. WPH was dosed at 1 µL with SPH dosed at 1 µL, 5 µL, 10 µL, and 50 µL to assess a dose-response potential. No significant difference in islet cell proliferation was seen between the 1 µL dose of SPH and the control. However, a progressive increase in proliferation was noted with the higher doses of SPH, with peak proliferation rates of 29%, 47%, and 57% observed at the 5 µL, 10 µL, and 50 µL SPH doses, respectively (see Figure 7). Only single assays were performed for this exploratory work, and therefore, statistical analyses were not possible. 

**Figure 8:**
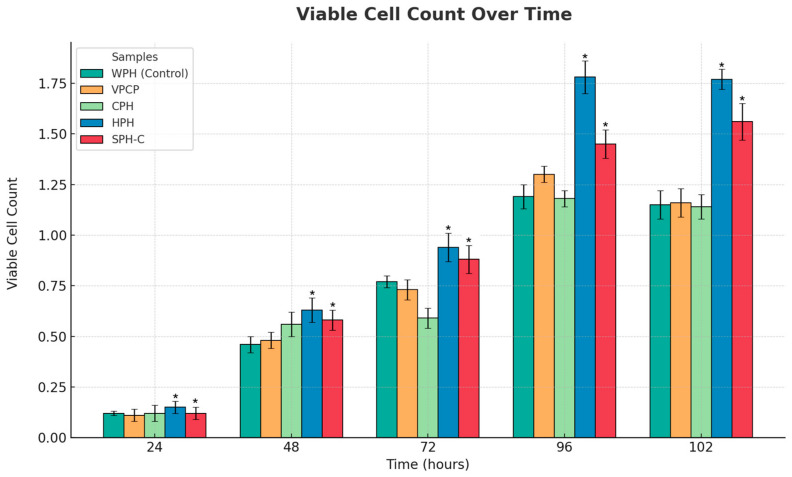
Effect of various marine protein hydrolysates and bovine collagen peptides on the proliferation of pancreatic islet cells (ABC-TC4286 hPIC cells) from 24 h to 102 h compared to control (WPH/whey protein hydrolysate), with all samples dosed at 10 µL/well. VPCPs are commercially available bovine collagen peptides; CPH is cod protein hydrolysate; HPH is herring protein hydrolysate; SPH-C is a salmon protein hydrolysate made with Coralase 7089. All concentrations of test solutions are 1 mg/mL at 10 µL/well. Each of the five conditions was analyzed in three independent experiments at five different time points, making a total of seventy-five assessments. Data points represent the mean ± standard error of the mean for each test article. * Indicates a *p*-value of <0.01 compared to control.

A second islet cell-proliferation assay was conducted to begin identifying the bioactive peptides that may support islet cell health (see Figure 8). Cod protein hydrolysate (CPH) and herring protein hydrolysate (HPH) were manufactured with the same enzyme sequence as employed to produce the SPH bioactive peptides. A new variant of SPH (designated SPH-C) was also included and was produced using a different endopeptidase (Corolase 7089) compared to the enzymes used for the CPH, HPH, and SPH. Additionally, a commercially available bovine collagen peptide mixture (BCPM) was evaluated against the control (WPH), with all samples dosed at 10 µL/well. No significant difference was seen between the control and either the bovine-derived peptides or the cod-derived peptides. In contrast, HPH exhibited a peak proliferation increase of 42% and SPH-C an increase of 32% compared to the control. The equivalent dose for SPH in the initial assay showed a peak increase of 47%. As noted previously, only single assays were performed for this exploratory work, and therefore, statistical analyses were not possible.

## 3. Discussion

This study was designed to provide deeper insights into the mechanisms underlying the pro-metabolic effects observed in clinical trials of a soluble protein hydrolysate (SPH) derived from salmon. These include reductions of 6% to 7% in body weight, driven by a reduction in adipose tissue, and improvements in glucose metabolism, as evidenced by moderate reductions in fasting plasma glucose of 3% to 6% [19,20]. Previous in vitro work demonstrated that SPH peptides possess potent dipeptidyl peptidase-IV (DPP-IV) inhibitory activity, as well as promoting direct glucose uptake into skeletal muscle cells [29]. Whilst these mechanisms could account for the moderation of plasma glucose levels with SPH, they do not explain the weight loss reported in two separate clinical trials involving overweight adults. Additionally, prior research has shown significant increases in serum adiponectin and GLP-2, prompting the hypothesis that SPH peptides might be activating other components of the incretin system, including GLP-1 [19,21,23]. 

Using cell-based assays, this study has confirmed that the peptides in SPH activate the incretin pathways of GLP-1 and GIP, which are critical for metabolic regulation. A dose-dependent response was observed in GLP-1 receptor activation, with both the 1 mg/mL and 10 mg/mL concentrations of SPH resulted in a more than 2-fold increase in GLP-1 receptor activity. Although only the 10 mg/mL dose reached statistical significance, a minimum fold change of 1.5 is indicative of a biologically relevant effect [30,31]. The GIP receptor activation by SPH peptides was more pronounced at the highest dose (10 mg/mL), showing a significant 3.8-fold increase. The two lower doses demonstrated similar effects, with just over a 1.5-fold increase compared to the control; however, these changes were not statistically significant. The laboratory standard GLP-1 and GIP agonists were dosed at a concentration of 1µM, equivalent to around 0.004 mg/mL, about twenty-five times lower than the lowest dose of SPH (0.1 mg/mL). 

Antagonism assays were conducted in triplicate using specific antagonists for the GLP-1 and GIP receptors, Avexitide/Exenedin 9–39 (MCE HYP0264) and GIP 3–42 (MCE HYP2542), respectively. No antagonistic effects were observed at any of the tested SPH concentrations (0.1 mg/mL, 1.0 mg/mL and 10 mg/mL) or with the laboratory standard GLP-1 and GIP agonists. Potential cellular toxicity was also assessed by means of cell line survival via nuclei count, and high viability (>80%) was seen in all assays. 

Following the fractionation of the peptides by molecular weight, GLP-1 receptor activity was localized within the smallest peptide fraction (<1000 Da). This finding aligns with previous in vitro assays demonstrating that DPP-IV inhibition by SPH is also concentrated in the low-molecular-weight fraction [29]. However, it should be noted that some of the peptides in this fraction were found to be between 1000 Da and <2500 Da when assessed by mass spectrometry (Table S1). The relative agonist activity of the commercially available GLP-1-based therapies, semaglutide and tirzepatide, was also assessed. The small peptide fraction showed a level of GLP-1 receptor agonist activity comparable to semaglutide, whereas tirzepatide showed nearly twice the activity. This observation is consistent with the clinical profiles of these two therapies, where tirzepatide is associated with a greater magnitude of weight loss compared to semaglutide [32]. 

The <2500 Da GLP-active fraction contains 1674 peptides, as determined by mass spectrometry-based peptidomic analysis. Thirty-seven peptides within this fraction have a DPP-IV inhibitory motif, wherein the motif is X1X2X3, in which X1 is any amino acid, X2 is proline or alanine and X3 is any amino acid except proline. Of note, proline in the second position from the N-terminus is a common feature of peptides with DPP-IV inhibitory effects [33]. Using the on-line predictive tool Peptide Ranker, a total of eighty peptides with a high likelihood of bioactivity, based on primary structure, were identified [34]. It should be noted, though, that this tool does not help to assess the likely type of bioactivity. Indeed, the specific peptide sequence responsible for this incretin effect within this peptide fraction is yet to be determined. However, given that this activity is confined to one specific peptide fraction, it is anticipated that the active peptides will be identified and sequenced. This has been successfully achieved by our group member Framroze to identify small peptides within SPH which upregulate the *FTH1* (ferritin heavy chain 1) gene system. This sequence was identified as EESGE, and the individual peptides containing this sequence are now being assessed for the modulation of tumor iron metabolism [35]. 

In contrast to the GLP-1 fractionation results, there was limited GIP receptor activity across the peptide fractions that were analyzed. There was a modest indication of increased activity in both the smaller peptide fraction (<1000 Da) and in the larger fractions (9000–10,000 Da and >10,000 Da); however, neither displayed notably high activity levels. In contrast, the complete mixture of peptides in SPH exhibited a 2.7-fold increase in GIP receptor activity, suggesting that the most active fraction likely resides between >1000 Da and <7000 Da; however, this specific range has not yet been tested. Tirzepatide again showed the greatest level of activity. 

The incretin hormones, GLP-1 and GIP, play a central role in metabolic health, increasing the effectiveness of insulin, delaying gastric emptying and increasing feelings of satiety. This has been exploited in the development of GLP-1-based therapeutics, which have shown marked benefits in facilitating weight management and the treatment of diabetes. Obesity is a significant health challenge of the twenty-first century, driven by a complex interplay of factors, including sedentary, time-poor lifestyles. Whilst a chronic imbalance between food intake and energy expenditure is one factor behind the obesity epidemic, other factors also appear responsible. Hyperinsulinemia in response to processed, high-glycemic-index foods is thought to promote increased adipose tissue and reduce periods of satiety; however, the data appear inconsistent [36]. Furthermore, dietary and physical-activity data from NHANES (the National Health and Nutrition Survey) did not fully account for the extent of weight gain observed in the US population from 1988 to 2006, suggesting additional factors at play [37]. Indeed, the adverse effects of environmental chemicals in the food and air are now becoming increasingly recognized as another contributor to weight gain and obesity [38]. Chronic exposure to small particulate matter (PM2.5) increases levels of oxidative stress and inflammation, disrupting metabolic signaling and exacerbating the imbalance between appetite and energy expenditure [39,40]. This results in adipose expansion in humans and animals, as well as impaired mitochondrial function [38,41]. 

Ageing further exacerbates the challenge of maintaining a healthy lean body mass, with changes in underlying metabolic activity, including a progressive decline in muscle mass, and increased systemic inflammation [5,6]. A recent large study showed that participants gained an average of at least 5% in body weight over ten years, with one-third experiencing a weight gain of 10% or more [3,4]. This pattern can be especially pronounced in women during the early years of the menopause, with a significant accumulation of body fat and a reduction in lean mass [42,43]. Even moderate weight gain in adulthood is associated with increased morbidity and mortality, highlighting the importance of maintaining a healthy body weight to support healthy ageing [4]. 

GLP-1-based therapies have been a breakthrough in the treatment of obesity, offering sustained weight loss and reduced cardiovascular risk [12]. However, there are no specific therapies for healthy, overweight adults, leaving dietary intervention and exercise as the primary strategies to prevent age-related weight gain. Nevertheless, the challenge remains for many of how to moderate the gradual weight gain of ageing. Previous clinical studies of SPH in overweight adults demonstrated weight reductions of 6% to 7% over 6-to-8 weeks of treatment at 16 g and 12 g per day, respectively [19,20]. The comparator, whey protein hydrolysate (WPH), showed no impact on weight, with a trend for an increase in BMI. Additionally, underlying biomarkers indicated improved health metrics, including a decrease in the pro-inflammatory cytokine interleukin-6 and an increase in serum adiponectin.

These are important health attributes of the peptides; however, a healthy metabolism also requires the maintenance of a critical mass of pancreatic beta islet cells. As noted previously, the peptides have been shown to significantly decrease *ALOX12* gene expression in vitro [17]. Raised *ALOX12* expression has been noted in obese subjects and diabetics and has been associated with the loss of pancreatic islet cells [26,27]. The islet cell assay showed no differential effect with the lowest dose of SPH (1 µL/well) compared to the WPH control (10 µL/well), with both WPH and SPH at a concentration of 1 mg/mL. However, a dose-dependent response was observed with SPH with a significant difference in islet cell proliferation from 5 µL/well upward of SPH. This is consistent with findings on islet cell health through GLP-1 and GIP agonisms, which stimulate and protect against apoptosis [44,45,46]. While published data have suggested that DPP-IV inhibitors can also protect islet cells, these findings have been inconsistent [47,48,49]. 

In diabetes, an important factor behind islet cell loss is chronic, low-grade systemic inflammation [50]. IL-1β and its associated inflammatory cytokines IL-6, IL-8 and TNF are implicated in islet cell loss in humans; however, other work has suggested a role of glucolipotoxicity as well [50,51,52]. Previous studies of SPH have shown antioxidant and anti-inflammatory effects, including a reduction in IL-6 in overweight individuals. These findings are likely the result, at least in part, of the upregulation of antioxidant protective genes and the downregulation of pro-inflammatory genes [6]. Whether the peptides’ positive impact on islet cell mass is, at least in part, via an anti-inflammatory mechanism is yet to be explored. However, such an outturn would be consistent with in vitro data, which demonstrated SPH to protect healthy human skeletal muscle cells in models of inflammation-induced muscle loss [23]. 

The second set of islet cell assays were undertaken to help identify the bioactive peptides that support islet cell health. Two other marine proteins, cod and herring, were hydrolyzed using the same sequence of peptidases employed in the production of SPH. Additionally, a commercially available beef collagen peptide mixture (BCPM) was assessed in the assays. The herring peptides demonstrated a similar capacity to enhance islet cell mass as SPH, with increases of 42% and 47%, respectively. In contrast, the cod peptides and BCPM showed no significant increase in islet cell mass compared to the control. A modified enzyme mixture was used to produce a different version of SPH (designated SPH-C), and this resulted in a reduction in islet cell expansion from +47% (with SPH) to +32% (with SPH-C). Nevertheless, this was still greater than the control, CPH or BCPM, which all showed an increase of around +20%. The findings suggest that the islet-protective bioactivity depends on both the specific mixture of peptidase enzymes and the protein source used to form the protein hydrolysate. 

There are some limitations to our study. Firstly, we did not identify the peptide fraction with the greatest activity at the GIP receptor, having used the same methodology as that to determine the peptide fractions containing *FTH1* and *HMOX1* activity. However, the results indicate that this is a distinct fraction from the one containing the GLP-1 receptor agonist activity. It would have been insightful to have used more graded concentrations of the peptides to more fully elucidate the pattern of dose response at the GLP-1 receptor, and work is ongoing to fully identify and sequence the individual peptides that activate the GLP-1 receptor. However, a bioinformatic analysis of the peptide fraction with GLP-1R agonist activity has not yet been conducted. The relevance of the impact of the peptides on pancreatic islet cell health needs further validation, including follow-up in vitro work with inflammatory media and in vivo models. Nevertheless, it is an encouraging initial signal, and previously published work utilizing pro-inflammatory and cancer media models employed higher doses of SPH, which protected human myoblast cells from damage with 100% survival rates [23,53]. Furthermore, despite these limitations, the study findings are consistent with the known effects of the GLP-1-based receptor agonism, which have shown improved pancreatic health in vitro and in animal models. 

In summary, we have demonstrated that peptides contained within a soluble protein hydrolysate (SPH) derived from Atlantic salmon (*Salmo salar*) activate GLP-1 and GIP receptors in cell line assays. These results are consistent with previous findings in clinical studies which have shown weight reduction in overweight, healthy adults, along with improved markers of metabolic health with SPH. Additionally, we present preliminary evidence suggesting that SPH supports pancreatic islet cell health in vitro, consistent with published data on GLP-1 receptor agonists. SPH’s profile suggests a potential to support healthier ageing, particularly in women undergoing the menopause transition. This period is often marked by weight gain, reduced energy, increased insulin resistance and an elevated risk of ill health, including cardiovascular disease. Based on comparative assay data with approved GLP-1-based therapies and digestion models, we estimate a potential minimal daily dose of between 1 g and 2 g for SPH. If validated in clinical trial work, this could offer a convenient and practical approach to enhance metabolic health in otherwise healthy adults. 

## 4. Materials and Methods

### 4.1. Assay for GLP-1 and GIP Receptor Activity

The activation of GLP-1 and GIP receptors in cAMPNomad-GLP1R and cAMPNomad-GIPR U2OS cell lines (CLS Cell Lines Service GMbH, Eppelheim, Germany, 300192) was assessed by Innoprot (Innovative Technologies in Biological Systems, 48160, Derio, Bizkaia, Spain), using their proprietary fluorescent sensors. These have been designed to enable the monitoring and assessment of signaling of G-protein-coupled receptors (GPCRs) in cell-based assays [54]. This is achieved by measuring second messenger signaling, which occurs upon receptor activation. In this case, activation of the GLP-1 and GIP receptors was measured by the level of cyclic AMP, using fluorescence intensity. 

On day 1, the cAMPNomad-GLP1R U2OS cell line was thawed, and on day 2, the cells were maintained in DMEM-F12 (Dulbecco’s Modified Eagle’s Medium, Sigma-Aldrich, St Louis, MO, USA, D8437) supplemented with 10% FBS (fetal bovine serum, Sigma-Aldrich F7524) at 37 °C in a humidified 5% carbon dioxide (CO_2_) atmosphere. On the third day, the cells were plated at a concentration of 30,000 cells/plate in a 96-well plate and maintained in DMEM medium supplemented with 10% FBS for 24 h at 37 °C in a humidified 5% CO_2_. On day four, the cells were incubated with the test compounds overnight and then diluted in OptiMEM (Thermo-Fisher Scientific, Waltham, MA, USA, 31985070), and compounds were tested in triplicate at each concentration. On the fifth day, the medium was replaced with 100 µL of PBS to perform the fluorescence intensity acquisition. For tFP650 detection, the filters 590/20 and 665/8 nm were used for excitation and emission, respectively. 

The test compound, SPH, was assessed against positive controls, a standard GLP-1 receptor agonist (Sigma-Aldrich H6795) and a standard GIP receptor agonist (Tocris, Minneapolis, MN, USA, 2257), both at a concentration of 1 µM (ca. 0.004 mg/mL). Negative control was PBS alone and sterilized water added to PBS. SPH was first diluted in sterilized water at 100 mg/mL and filtered with a 0.2 µm filter to avoid contamination. A dose-response assay was performed at three concentrations in 1:10 serial dilutions of SPH, starting with 10 mg/mL, consistent with previous work undertaken to assess the *FTH1* and *HMOX1* activity of SPH. 

Red cAMPNomad biosensor’s fluorescence intensity was quantified in the cytoplasm of the living cells using the following approach. Nuclei were stained using Hoechst dye (0.5 µg/mL) for thirty minutes, and fluorescence was measured using the Cell Insight High-Content Bioimager (Thermo-Fisher), using filters of 380/10 and 460/10 nm for excitation and emission, respectively. To detect the fluorescence intensity of the red cAMPNomad biosensor, the filters used were 549/15 and 640/30 nm, respectively. For fluorescence quantification, Cell Software, version 2.1.1 (July 2022) (Thermo-Fisher) was used. 

The abovementioned methodology was also used to assess both GLP-1 and GIP agonist activity, and more information regarding Innoprot’s proprietary cell lines and immunofluorescence methodology for assessing specific receptor activity, using immunofluorescence, was published previously in more detail [45].

### 4.2. Peptide Fractionation

Peptide fractionation was undertaken by Creative Biostructure (45–1 Ramsey Road, Shirley, New York, 11967, USA). Five lots of SPH of 1 g each were dissolved in 10 mL of PBS buffer (pH 7.4), respectively. Dialysis was undertaken according to dialysis bag-size groups of 1000 Da, 2000 Da, 3000 Da; 3000 Da, 4000 Da, 5000 Da; 7000 Da, 8000 Da, 9000 Da and 10,000 Da (with ±500 Da accuracy). The first dialysis of the SPH mixtures was performed in the different groups of dialysis bags for 12 h, using PBS dialysis buffer, pH 7.4. The dialysate was collected separately for the second dialysis, which was performed for 12 h with PBS dialysis buffer, pH 7.4. The dialysis was again performed using the different-size dialysis bags as per the groups of bags described above. The external dialysis fluid was collected from the different dialysis bags, and lyophilization of each was undertaken to prepare lyophilizates of different molecular weights, which were then labeled. This resulted in peptide samples of peptides <1000 Da, 7000–8000 Da, 9000–10,000 Da and >10,000 Da, confirmed on both concentration and HPLC (high-performance liquid chromatography) testing. 

### 4.3. Pancreatic Islet Cell Assay

The pancreatic islet cell assays were undertaken in triplicate by Hofseth BioCare. ABC-TC4286 Human Pancreatic Islet Cells (hPIC) from Accegen Biotechnology (Fairfield, New Jersey, 07004, USA) were seeded in a 96-well plate at a cell density of 10,000 cells/well in 200 µL cRPMI-1640 growth medium/well with 10 µM Reg 1alpha protein (transfection supernatant). The cells were incubated for 102 h at 37 °C. SPH bioactive peptides were dosed at 1 µL/well, 5 µL/well, 10 µL/well and 50 µL/well to assess for potential dose-dependent response. Whey protein hydrolysate (WPH) dosed at 10 µL/well was used as control. The proliferation response was measured every 24 h using WST-1 (Roche) method. The OD450 value was read on a Tecan GENios FL multi-detection microplate reader, where the observed absorbance directly correlates to the number of viable cells/well. The OD450 reading is proportional to the number of viable cells. SPH doses were selected to try to attain minimally effective levels and then assess for dose response. Prior work with SPH in inflammatory and cancer models used up to ten-fold higher doses to account for the adverse environment for cell survival in these media.

### 4.4. Liquid Chromatography–Mass Spectrometry Peptide Sequencing

The peptides were resolved with a loading buffer of 2% (*v/v*) ACN and 0.05% (*v/v*) trifluoroacetic acid. The peptides were loaded into a trap column (Acclaim PepMap 100, C18, 5 µm, 100 Å, 300 µm i.d. × 5 mm, Thermo Fisher Scientific) and then backflushed with a loading buffer (described below) onto a 50 cm × 75 µm analytical column (Acclaim PepMap RSLC C18, 2 µm 100 Å, 75 µm i.d. × 50 cm, nanoViper, Thermo Fisher Scientific, Bremen, Germany) for LC-MS/MS analysis. 

Conditions for ultra-high-performance LC were as follows: loading pump, flow rate of 20 μL/min with loading buffer; 2% (*v/v*) ACN and 0.05% (*v/v*) formic acid (FA) and nano/cap pump, flow rate 0.3 μL/min with a gradient of two buffers, A and B. Buffer A was 0.1% (*v/v*) FA, and B was 80% (*v/v*) ACN, 0.08% (*v/v*) FA. The LC gradient was run for 120 min, from 3.2 to 80% buffer B. Peptides from the 12 most intense peaks were fragmented, and the mass-t-charge values of these fragmented ions were measured (tandem mass spectrometry, MS/MS) with a Q-Exactive Quadrupole-Orbitrap mass spectrometer (thermo Fisher Scientific, USA). 

The Q-Exactive mass spectrometer was set up as follows: a full scan (300–1500 *m*/*z*) at R = 140,000 was followed up by (up to) 12 MS2 scans at R = 17,500, using an NCE setting of 28. Singly charged precursors were excluded for MS/MS, as were precursors with *z* > 5. Dynamic exclusion was set at 20 s. Finally, peptide identification was performed using MaxQuant, an integrated suite of algorithms specifically developed for high-resolution, quantitative MS data. 

## Data Availability

The original data presented in the study are included in the article/Supplementary Material; further inquiries can be directed to the corresponding author.

## Supplementary Materials

The following supporting information can be downloaded at: https://www.mdpi.com/article/10.3390/md22110490/s1, Table S1: Oligopeptides of Low Molecular Weight GLP1 activating fraction in SPH.
