# Supplementary material for: Initial Exploration of the In Vitro Activation of GLP-1 and GIP Receptors and Pancreatic Islet Cell Protection by Salmon-Derived Bioactive Peptides
Source: Mar Drugs. 2024 Oct 30;22(11):490. doi: 10.3390/md22110490 (PMC11595994; doi:10.3390/md22110490)
Supplement: Supplementary file 1 [file marinedrugs-22-00490-s001.zip › marinedrugs-3220309-supplementary.pdf]

Table S1. Oligopeptides of Low Molecular Weight GLP1 activating fraction in SPH

Sequence	SEQ ID NO	Mod. Sites	Mass	DPPIV motif
TADQLEDFKEAFGL	1		1583.76	
NWDDMEKLWHHTF	2		1758.76	
KMPGEAGLLGYTEDQVVST	3		1994.97	
KGSPELSDLTEQLGETGKS	4		1975.98	
KNSYEEALDHLETL	5		1661.79	
EVLEKEAADLLTEK	6		1587.85	
KATKLEQQVDDLEGSLE	7		1902.96	
LGSDLVDHEDYKFGHTK	8		1960.94	
SEVLDMLKDAMLAK	9		1563.81	
GFLEEEELKFVLK	10		1580.86	
VVDQEKLDHFM	11		1360.66	
KLDSELPVDEVFGH	12		1584.79	
EGVDDLVQEARGQ	13		1415.67	
LVTWYDNEFGYSNR	14		1763.80	
FDMFDTDGGGDLSTK	15		1605.67	
LTPDQKKELSDLALK	16		1698.97	
HFADNLKDWSKVVL	17		1671.88	
DVLPAVSTGVDLQDL	18		1541.80	
ALEEAEGTLEHEESKLL	19		1897.94	
ELAGAELLDVSERVQ	20		1628.84	
LLTNWDDMEKLWHH	21		1837.87	
PQSYEEALDHL	22		1301.60	
PMNSLLNAADLK	23	2,Ox.[M];	1302.67	
KAVLHDNYHLLEGL	24		1621.87	
LTNWDDMEKLWHH	25		1724.79	
LTEDGEFGRQL	26		1264.62	
SEAEQDQLLADFH	27		1502.67	
LTGDQLGDLYK	28		1222.63	
NVPDQKKELSDLALK	29		1697.95	
KAVLHDDYHLLEGL	30		1622.85	
KGPSELSDLTEQLGETGK	31		1888.95	
HFADNLKDWSKVV	32		1558.80	
GLPAMSPQLDDLNQVAKASH	33		2092.06	
MLDQDDWAAWTKF	34		1626.72	
STGVFTTLDKAGTHLQ	35		1675.86	
LLQSAETEKELANMK	36		1704.88	
TYTTKEDVVDTVGLR	37		1696.87	
VSRLYLPNDNFFEGK	38		1798.92	
SEELADNLALNDM	39		1434.64	
MLDQDDWAAWTKF	40		1626.72	

Sequence	SEQ ID NO	Mod. Sites	Mass	DPPIV motif
GLVMDFKNKYEDELNKR	41		2099.07	
EVQHRLDEAENL	42		1452.71	
NVKKPVYDEEVVGQY	43		1766.90	
VDEEKLEWDKAWDV	44		1761.83	
GPGLQSMFDFQVK	45		1453.71	
EAANAELELK	46		1087.56	
VVGDDLTVTNPKRLQQ	47		1782.97	
PDELESSQKEARTV	48		1588.78	
KKVNGVTPASYDALKKVVK	49		2045.21	
LTGDPAKKELSDLALK	50		1698.96	
WDNDMEKLWHTH	51		1611.70	
ELKTLDDLEDELYAQ	52		1794.86	
DPADTEKELANMK	53		1461.69	
EVPSLPQDLHLV	54		1346.73	
EVVLDVPGPVKNL	55		1378.80	
GYMGPDQLDAALK	56		1378.67	
EHRDPANLKWGDAGATY	57		1900.90	
VLGNSGLSEVLHMT	58		1456.74	
ELGPDVDVNLPK	59		1295.68	
SNPVPYWEVQPLTFR	60		1832.94	
TVPRPNVSVVDLVTR	61		1651.95	
GLDASSGELPVVGLR	62		1469.80	
KTLEQQVDDLEGSLE	63		1703.83	
KNEAEGTLEHEESKLL	64		1826.90	
GDAGTLLGVEYKSFLK	65		1697.91	
GEVEDLVQEAR	66		1244.61	
DPDSGVLVSRMLDFVKKKH	67	11,Ox.[M];	2187.15	
SGGTTVYYALLLRFYSYHE	68		2240.10	
GFTNEEKLGLY	69		1270.63	
PDQLTLGDLYKSF	70		1496.76	
EGSGYRGEAEVELK	71		1523.72	
FDMFDTDGGGDLSTK	72		1605.68	
PDPAFHEELTKEEPD	73		1753.79	
ETRELEEAKKKLAQR	74		1829.03	
KDAAYLEELQGNPKF	75		1722.87	
PEEMVELCFKPEEMVEYK	76		2230.02	
LVPEGQFMDNK	77		1277.62	
KTALEKAGYPDKLLLST	78		1848.03	
DSEEEFPDLSLH	79		1417.60	
KDYLEELQGNPK	80		1433.73	
EVTMDRLLQTM	81		1336.66	
EVQHRLDEAENL	82		1452.71	

Sequence	SEQ ID NO	Mod. Sites	Mass	DPPIV motif
VVDQYEVLKLK	83		1333.77	
HFLFDKPVSPL	84		1299.71	
GVGGSVQHSSVPVWSGVGVQ	85		1922.96	
PLEEVGDPLEEVVEAK	86		1752.90	
GSGEAEVELK	87		1018.51	
MFGTGVLKQDPEDVLSV	88		1834.93	
PDQKEELALKLVAK	89		1581.91	
LLTENGEFGRQL	90		1376.72	
WDDMEKLWH	91		1259.55	
KEGLKGADPEDVLVSL	92		1669.90	
GFTNDEKLGLY	93		1256.62	
KNSGLGDPVTLPLVVK	94		1636.95	
KDSYGQVGDEAQSKRVAL	95		1950.99	
TAGGAKEELLSWVLEEANAT	96		2089.04	
LDDLSSNMEAVAK	97		1392.67	
GNSGLTDVLHML	98		1256.63	
LQTENGEFARLQ	99		1405.70	
VLHDNYHLLEGL	100		1422.74	
MADTFLEHM	101		1094.47	
PQGLEVLVPHP	102		1185.66	
SYEKLPDGQVLTGL	103		1519.80	
ELEEELEARAE	104		1317.61	
FHDNVLLFLNDHFVKL	105		1971.06	
GVATDVVRGPGGPLWQVLSPTPN	106		2317.24	Y
KDPLLYGAGSALALDVVTLPNLGM	107		2428.32	
WLTKQEYDPCGPSLVHKR	108		2157.11	
VPGPLDLKVPVVDVT	109		1547.90	Y
TAALPTAFPADQL	110		1315.69	
KPVEFTAWQKVDLAHAM	111		1971.02	
PQSYEEALDHL	112		1301.60	
KNGGYKKEQETREEQVGWLDP	113		2491.23	
LAEEAEGTLEEH	114		1327.60	
KEYLDEALTSLK	115		1409.75	
KFAVLDPEATGFLK	116		1535.85	
GEGATLEHLQQGQCLL	117		1696.84	
WDNDMEKLWHTH	118		1611.70	
LAEEADRKYEEVARKL	119		1920.02	
KFVEEEYPDLTKH	120		1634.80	
PHDEEEFPDLSLH	121		1564.68	
NSYEEALDHL	122		1190.53	
EAANAELELKLR	123		1356.75	
QLSAETEKELANMK	124		1591.80	

Sequence	SEQ ID NO	Mod. Sites	Mass	DPPIV motif
AAEDKSKQLEDDLVAL	125		1744.89	
LGDEQKGDGMLGLDEF	126		1723.78	
QSEKLQQFFDTH	127		1507.72	
KFVQVEEEYPDLTKH	128		1861.95	
WDNDMEKLWHTH	129		1611.69	
KDLLDPLLSDR	130		1284.72	
KAADYLEELQGNPKLF	131		1835.95	
LLYADKFQRYK	132		1444.80	
RPLEEVVEAK	133		1169.64	
KHLVTFFAYLKDDLLYAQ	134		2185.17	
WDDMEKLWHH	135		1396.61	
GFTNDEKLGLY	136		1256.62	
TVGTPRELPTEKVF	137		1573.86	
KFVEEEYPDLTKH	138		1634.81	
ELELSTAQGGLEAGGAT	139		1603.78	
KEAWDDMEKLWH	140		1587.72	
DLKVLPAED	141		999.54	
HGGVDEDLYKFGHTK	142		1702.82	
GLVLDSGDGVTH	143		1169.58	
RSTELGKLGVDQMVAT	144		1704.90	
AEQDQLLADHF	145		1286.60	
LFAPTNDAFALK	146		1307.70	
KDAHGWNSVPVWSGVN	147		1752.85	
FSDEEEFPDLSL	148		1427.62	
NDEELNKLVQVT	149		1401.72	
ELGEDMEYQFR	150		1416.61	
VESSLVWVVLASH	151		1425.77	
EFVDLLNAKQ	152		1176.63	
QVDLDPNLQLVRTH	153		1647.88	
LAQSDELALEEAKKKL	154		1786.00	
FHTLGFASK	155		1007.53	
QVDPMRENVAELK	156	5,Ox.[M];	1544.78	
THYQVASVESSSWAMHVGK	157	15,Ox.[M];	2120.00	
SVLPEGQFMDNK	158		1364.65	
KSLVEDEQSLQ	159		1275.64	
TLPDQKKELSDLALK	160		1698.96	
STELFKMK	161		983.52	
LAPSDENWFYTR	162		1498.69	
VSPWEKAMKGN	163		1246.63	
GVAHDEDLKLVGY	164		1415.72	
RMLEEALGRSAKFAGK	165		1763.95	
GPGKVALQFALLQ	166		1341.79	

Sequence	SEQ ID NO	Mod. Sites	Mass	DPPIV motif
EVQHRLDEAEPE	167		1451.69	
KNGPDLDVNLPK	168		1309.70	
PPAAPEFLTPLFNAH	169		1621.84	
FVQVLDTELKVQVQ	170		1645.92	
QPESYVLVPAALFWDAATL	171		2091.07	
KNKAGYVPKELLGM	172		1547.85	
EGLEMHEAEVELK	173	5,Ox.[M];	1529.72	
SELEEELKTVTN	174		1391.69	
HVARHDVHDKLRQW	175		1796.95	
SFEPVWALGTGK	176		1291.66	
KACDALLQEQESHYGPT	177		1889.87	
FTPDGNVDDTAAFTFGL	178		1787.81	
GLDGLELVAHLPSP	179		1417.76	
KGVLGLPELNGKLT	180		1438.86	
GLDGLELPTEK	181		1171.62	
PASTNKTMNVDTPASAL	182	8,Ox.[M];	1733.85	
GLETELVDASKTLDP	183		1587.82	
LYETPTGWKF	184		1241.62	
ETGVADKLAY	185		1066.54	
KPEFFHTLGFASK	186		1508.79	
KELEKSEYKMELDDL	187		1869.91	
KGPDGDLEEELKNVTN	188		1757.85	
GLTADEEYEGFLK	189		1471.69	
KELSPGCSQNKLPAPDEQKL	190		2182.13	
KGVLGLPEVYGTVQAP	191		1627.91	
VQPVASMLSEKMELT	192	7,Ox.[M];	1678.85	
HLVDEPQNLLK	193		1305.72	
DDMENWKLWHTH	194		1611.70	
KGSLEGPDLDVVQPKA	195		1652.89	
VPMLVGNDELDEML	196		1574.75	
LVRYPNDNFFEGK	197		1598.80	
EVQRDTLDYLMK	198	11,Ox.[M];	1526.75	
SEAELVDYKNEHDSLQVVP	199		2172.05	
GVATDVALQPGGPLGQGLLQVDPN	200		2316.24	Y
QQNHSDLEKALNTL	201		1610.80	
PQAGAQAEFALKENDGAT	202		1817.87	
GSDGAQEAVLR	203		1102.55	
VSVVDLTVR	204		987.58	
EVATRQPLDDLNQKHTGL	205		2035.04	
GFTNEEKLGLY	206		1270.63	
KDQRKNLQEELSDL	207		1715.89	
FSVVPMEKLDHFM	208	6,Ox.[M];	1595.76	

Sequence	SEQ ID NO	Mod. Sites	Mass	DPPIV motif
KNPMQGSLEDQLLA	209		1543.78	
GLTEGADYYLR	210		1257.62	
LSEEMLNEKF	211		1239.60	
GLFRPTPNVSVVDLLAAT	212		1870.06	
LVDVPVGEELLGR	213		1395.79	
SELRDLLKTM	214		1205.65	
GFAQEELNGLYK	215		1368.68	
VLTVGSQVSNKVNQ	216		1472.80	
VLDKTWMDVLAPELK	217	7,Ox.[M];	1773.93	
DLDLRKDLYAN	218		1335.69	
VLGNPSDEDMAGKRL	219		1601.80	
GYDLLLVPEPKLL	220		1469.86	
LHEWLVEWMKTL	221		1584.84	
TVRDLLDDTEYKEY	222		1759.84	
ELEEELEAEAAR	223		1388.65	
PLLFVLVPGPPVSVKF	224		1709.04	
NVLSGGTTMYYFAKCK	225		1782.85	
KKDLDDLELTL	226		1302.72	
GVLEENMRNKLASL	227	7,Ox.[M];	1589.82	
EEELTWEEELKTV	228		1634.79	
KVELVLDYKPTLM	229		1548.86	
LSTELFK	230		837.47	
VEEDALQRAKQD	231		1401.69	
TVRLPVYDAEVV	232		1360.75	
KNEEELKTVTN	233		1304.66	
TGGLHETAYNSLM	234		1393.64	
GFLLTMFWSGVLQKSSTF	235		2049.06	
LFADNKDLGCKVVL	236		1534.83	
SPGAVGQVAANNALL	237		1381.75	
DSYEEALDLH	238		1191.52	
GVNGWMLTMFGPVEVLW	239		1935.97	
ESFAEKMLAVHAL	240		1445.76	
LFTEDEDVTGK	241		1253.59	
LYEEELREL	242		1193.61	
LFDSDGGGDLSRT	243		1339.61	
VLMFPGDDDNVLYVEGL	244		1895.90	
KVEASGPLDYYVNQPTAAVP	245		2119.05	Y
PATQVGVKGLRWL	246		1424.83	
KDQSLGELLKTL	247		1344.77	
KGLLLEMCDKSLLLGLDNGVPQ	248		2356.24	
KDYPVSLEEEPFK	249		1580.79	
GLPEEHPTL	250		992.51	

Sequence	SEQ ID NO	Mod. Sites	Mass	DPPIV motif
ALGGMTPGLPGLFTVPGGAL	251	5,Ox.[M];	1841.98	
VLRVHGESEWPMYNKF	252	12,Ox.[M];	2007.99	
HLVNDPNKGTFDDYVEGL	253		2032.96	
LAPYDTPLYSNVQLPMPTL	254	16,Ox.[M];	2149.08	
APPPLDLSSLK	255		1137.65	
VPDAVYPPAPKAGLGN	256		1565.84	
KLEDATFKLDY	257		1342.69	
EEANSKLELQLR	258		1429.77	
KKNYELTGPGFSQKFYT	259		2008.02	
LGGDHFVKL	260		985.55	
TFPGLDLPKML	261		1231.68	
QGAEGDLSLGLK	262		1187.63	
TLHLMLPSPVTAQ	263		1407.77	
EGGGLGVRDLHGYVD	264		1543.76	
KGNGKLLKMGHTLWQ	265	9,Ox.[M];	1726.93	
RELEELSER	266		1160.59	
GEAVGETYQVKLW	267		1479.75	
TVPRPNVSVVDLL	268		1408.82	
SPADAVGLVTPMF	269		1304.66	
KEPQEAADLLTEK	270		1471.77	
GPPGPPGSMPWVGPQDPT	271		1773.82	
TESFGPGTLPKNPAH	272		1552.77	
LGGGVLFGWGPVEGMLPDL	273	15,Ox.[M];	1929.96	
AAFPPDKPQSDYKQ	274		1591.78	
KDETEVELEQR	275		1375.68	
EEELDRAQER	276		1274.60	
KGLVELNKPGVDAL	277		1452.83	
LGRPEEHPTLL	278		1261.69	
SSDLKDDLSLR	279		1248.64	
QETLLGETGKGPTQPV	280		1654.86	
QVAPGPAGEDGKVYGMVSPSQPVGG L	281		2497.22	
EVCHLKRALEE	282		1326.69	
PGGETKTPAGTWKANPLVMH	283		2092.06	
KGEVLPGVDAL	284		1097.62	
VPEEVAVSVYMGLGN	285		1563.77	
DSYEEALDLH	286		1191.51	
ALGNPLPDLVGLRT	287		1435.82	
GFAGDDAPRLGFPS	288		1406.67	
EVDTLQSVGDDLTNTL	289		1719.82	
GELLNTVGR	290		958.53	
RFDPENDKWLR	291		1475.74	

Sequence	SEQ ID NO	Mod. Sites	Mass	DPPIV motif
KFDLKHDGDPLVLLEPVLAH	292		2256.23	
DPVHRDLEAENL	293		1407.68	
KGELKQDPEDVLVS	294		1556.82	
APGAVDLVNLSVPA	295		1322.73	
QELQVDDLERTEEQKKLR	296		2257.17	
KFDLSPDDPWSKFPMQL	297		2051.01	
LGGDHFVKL	298		985.55	
GPQPPEPLFVSK	299		1295.70	
SYEEALHDLETL	300		1419.66	
VVDQEKLDHFM	301	11,Ox.[M];	1376.65	
EVDDVYRSGTLLKPN	302		1705.87	
EGGLYEHVLQEGPV	303		1526.74	
TFLLDQHGLTFTV	304		1491.78	
LEEELGAK	305		888.47	
PDEDGEFRLGQ	306		1262.56	
PADLAKHAVKVSEQTKVTKY	307		2213.24	
LLQSAEVMQELANM	308		1576.78	
VLPLVEEPLLPDYLPEVPSH	309		2256.23	
GLVFNALEADLVGLR	310		1586.90	
EVAPGQEYHTLGTGQ	311		1586.75	
EGSGYRGEAEVELK	312		1523.73	
NELDDLKGPA	313		1071.53	
STRPPGLPGYNWGVTFFRGAP	314		2277.18	
PPCRTKYETD	315		1209.55	
PGGLEEVGSEVASF	316		1377.66	
KEKEYKNHTVLEGSLEQEKK	317		2417.26	
GTNQHPANLKGWDQ	318		1565.75	
EHSPAMALPELM	319		1325.63	
KANLSSDLALLNAKQ	320		1585.90	
VNDLFEPNLAAF	321		1349.67	
EPVPNPDGDHDLKRT	322		1689.81	
ESAEQELKQGKFDE	323		1637.77	
KAVWFDLVFTAK	324		1424.80	
KNETEVELEQR	325		1374.68	
GPRDQKYEEGQAELE	326		1748.81	
ELEELSELR	327		1117.57	
EYYNRLPELK	328		1324.69	
KVAESGPLNTGTRNF	329		1590.84	Y
YSGDGAQEAVRL	330		1265.61	
GPFALELELSERKNPC	331		1802.91	
KLCAGDLPHPEFVDLLNAK	332		2080.08	
KDLLDPLLSD	333		1128.62	

Sequence	SEQ ID NO	Mod. Sites	Mass	DPPIV motif
KGLGPCEELNKLLGKE	334		1727.92	
PLSPVLNYLLGAL	335		1369.81	
DDMEKLWHHTF	336		1458.65	
KQGHDPTFQLK	337		1298.67	
KELEGGTGLQWTNEEKLG	338		1989.00	
KLGSLRTPLVGEL	339		1382.83	
EDLGAGLNQALQHDYAT	340		1815.87	
KNVLPEKFQHLL	341		1465.84	
KDTFTKFDELQEPET	342		1827.88	
PEFVDLKALLN	343		1258.70	
KVNGPTLDENQVKAL	344		1625.87	
ELGPDLDVGKAAPV	345		1380.74	
GLPLLKARWQAQ	346		1380.82	
KANEKPVDLLNAKQ	347		1567.87	
EDVTKLVPDEVFGH	348		1584.79	
LAEKMLSCALEEVAKRL	349	5,Ox.[M];	1920.02	
GPLEYFPRNPNESKTGPLM	350		2147.06	Y
KGVQHDTFLEMH	351		1441.69	
WNDDMEKLWHH	352		1510.65	
SEVPLPVGKLF	353		1185.69	
TVQDELNFLR	354		1234.64	
DVGPQFNPDFPLKNT	355		1688.83	
WAGLEDRPEASV	356		1329.64	
KDCKVGDLLFKR	357		1421.80	
GVAQDDPLLVYH	358		1326.67	
PALYMDLPAVSEK	359		1433.73	
QELQVDDLERTEEQKKLR	360		2257.17	
PSQLVWSMESLLTVF	361		1736.89	
GPGKMQGSLEDQLLAANPL	362		1939.00	
EEANSKLELQLR	363		1429.76	
EPMKYPTVLTVPQ	364		1502.79	
TTPRYGENGEFGRQL	365		1724.85	
CPNEYGLGLPLETR	366		1561.78	
APAPLGPVNSQLL	367		1276.73	
KFEVSWPDDPSYLVEQL	368		2052.01	
LVPELDGKLT	369		1084.63	
KNGPDVDVQVPAK	370		1366.72	
KPPYDKLEDMMPQ	371	11,Ox.[M];	1607.75	
GPLLEGRQVVDLT	372		1396.78	YY
EELSRPHHEA	373		1204.57	
KGVAVLEPYMPDLQ	374		1559.81	
WHTKYKQEGTWHVVGHPL	375		2203.12	

Sequence	SEQ ID NO	Mod. Sites	Mass	DPPIV motif
ALDTLDEGFKGK	376		1293.67	
KKNYELTGPGFSEPG	377		1623.79	
KGSPGLFGFYAKAP	378		1439.76	
LAVRNDEELNKLL	379		1526.85	
ESSWTTFPMLSQ	380		1413.64	
GLTEDATYEFH	381		1282.56	
TLKDGEFGRLQ	382		1263.67	
LGFAGDDAAPR	383		1089.53	
EFHELMMRQ	384		1220.56	
EGGVFTTLSVAA	385		1151.60	
DREYYMKVLENR	386		1615.78	
PTPAFLPPTFTGW	387		1431.73	
KNLLEKPLGLF	388		1271.76	
KMLVQPEDYLMKLL	389	2,Ox.[M];11,Ox.[M];	1752.92	
HMNDMEKLWHH	390	2,Ox.[M];	1493.63	
KKDLDDLELTL	391		1302.71	
TVALGWVAGPGGPKSDLVPN	392		1935.02	
EVAPGQEYEFR	393		1324.62	
ELEELSERASNGL	394		1446.70	
LPVEEPLLDPGDQELAVPL	395		2044.07	
GTGDELKGLY	396		1052.53	
EVAGRDLTDYLMKLL	397		1736.92	
EDLGAFTLAFKPGDYAT	398		1815.87	
EVGARAEETLEAT	399		1375.67	
YPDNTTFPMTV	400		1285.57	
WNDDMEKLWHH	401		1510.65	
KSCELKDWSKVV	402		1421.74	
VGATDVVQNLKLAGPGAVLSVVPN	403		2318.30	
GPAGGPVLFGVAGPGGPLGQATNPA	404		2159.13	Y
KEVLAGKGPSLVHKR	405		1618.99	
KGSTADESESEDLLGSQR	406		1908.89	
PSLHFMTQLM	407		1204.58	
KGVASYDLEPPDHHPLGPQL	408		2170.10	
PGASLWVSMESLLT	409		1490.75	
GQELMLGNDEEELNAELT	410	5,Ox.[M];	2020.92	
ALAGLKGDLAGHYY	411		1448.75	
GNSGLTDVLH	412		1012.51	
LGGALSMVVLDKL	413		1315.76	
KACDALLQEQESHLMGT	414	15,Ox.[M];	1889.86	
VDEEKLWGDK	415		1218.60	
AANAELEKL	416		958.52	

Sequence	SEQ ID NO	Mod. Sites	Mass	DPPIV motif
GPGSAPEGLSEFLKQ	417		1516.77	
AFLGWETVKKYGPSP	418		1679.87	
SDLALKLVAK	419		1057.66	
LFDSDGGGDLSRT	420		1339.61	
GFQRLKDHVDQFTTSDL	421		2006.99	
LVNKEDGQLTRAL	422		1456.81	
ESNGEEQEAKAE	423		1320.56	
EGDPLEAVRTQ	424		1214.59	
KGVAVLPELNGKLT	425		1438.86	
FVDLLNAKQ	426		1047.58	
GPPMGGSGVGMPRGPPGPPG	427	11,Ox.[M];	1774.82	
KAADTFNFKTF	428		1289.65	
PVERQCALELLK	429		1398.79	
LEYSKELTWWLVPV	430		1762.93	
LLTNGEDDMEKLMSEL	431		1837.85	
VPDPQMGPVPHPVTGY	432	6,Ox.[M];	1706.83	
LLTEDWFNVVAA	433		1377.70	
KSDPKFQPYVL	434		1321.71	
KNDKKAEVPPEEEVKAK	435		1939.05	
AENAARKAAGCGLPAAPG	436		1624.81	
NDALHLDDVTHLE	437		1491.71	
HFEGDVRLQ	438		1100.55	
PGVPKYVDLYANL	439		1448.78	
FVQRVVEKL	440		1117.67	
VDWTDAESK	441		1050.47	
ALGDRAYAGTVDYNLT	442		1699.83	
LVPEGQFMDNK	443		1277.62	
VDASRLDGL	444		945.50	
PHPLKEGDQLASQY	445		1582.79	
TVYEEALDLH	446		1189.57	
KEGLKQDPEDVLPPNHRT	447		2073.07	
EACHGTLHEEEAKLL	448		1679.81	
EVVLDVPGPVKS	449		1238.70	
EPADTLHTPDLVRAT	450		1635.84	
LGFAGDDAAPR	451		1089.53	
AEGWKQKAEGTLEEH	452		1712.83	
KPELQNLDGYVKGLE	453		1702.91	
NSYEEALDFVFVT	454		1533.71	
HLVNDPNKGTFDYSQEGL	455		2033.96	
VLAYFYEAQEEVQGLYK	456		2050.02	
YSPLLDDRKLK	457		1347.77	
ELRLKQEYFVGL	458		1494.83	

Sequence	SEQ ID NO	Mod. Sites	Mass	DPPIV motif
ESRLLQELSELR	459		1472.79	
KFADVLCDLVPQ	460		1347.71	
GDAYEVLKLK	461		1135.64	
SFDTPSCQWLHLDDGLR	462		1989.89	
KSENGEFGQRL	463		1264.64	
TVDASAEHELSDLALK	464		1698.85	
KLDENHEEFLK	465		1401.69	
ESKLTDVNFASCLGPGP	466		1734.83	
GPAMQLSRWAELVQL	467	4,Ox.[M];	1714.90	
KYALYGDNWSFLK	468		1604.80	
TTRTLAAAWEQQKQEAVLR	469		2200.17	
EATKPQKTGEEAKKKLAQ	470		1985.11	
SEPLYSNVLHTVNTM	471		1704.84	
TVKLAEGTLEEH	472		1326.70	
ELEEELKT	473		990.50	
ATGDFKNKYEDELNKR	474		1927.96	
GTDDVHDLLLFAKVHASGF	475		2042.03	
GSGGVCFDEKSNGM	476		1387.56	
FLGMESAGLHET	477		1291.60	
EGVDDLVQEAR	478		1230.60	
VLLEGDLETR	479		1144.62	
EVDTLLMTLDDL	480		1377.67	
LELLEKAVP	481		1011.61	
KGVAVLPELDGQHKH	482		1627.91	
ETSPDSTELFK	483		1253.60	
AFPPDKDVGNAVYQ	484		1520.74	
LHLDDAVRGL	485		1108.61	
PQEYEEELKVL	486		1376.69	
LTFSYGRAL	487		1027.56	
CAPNKTELLSWVLEEAGGA	488		1987.99	
GPAGGPVLYGVAPGPNLNGAP	489		1874.97	
EACHGTNKHEESKLL	490		1695.81	
WLFKFYKMEYK	491	8,Ox.[M];	1598.79	
DPWKPAEETLSNT	492		1487.71	
LEELSERL	493		988.53	
VPDGLGLAQAPVYPCKGLEAPA	494		2166.13	
FMPGLADMR	495	2,Ox.[M];	1053.49	
GFAQEELTWSAL	496		1351.66	
TEELVDASERRLL	497		1530.80	
LTMYPGDTELK	498		1267.62	
KGYLQEAEDLEEK	499		1551.76	
EVEVNMMTEEVVEAK	500	7,Ox.[M];	1752.79	

Sequence	SEQ ID NO	Mod. Sites	Mass	DPPIV motif
KSPELVLLQTLVLAH	501		1661.00	
TVPHELTGPGKKNDNP	502		1703.89	
GEGALEYFLGEQGL	503		1482.71	
EYQNCYEVLKLK	504		1529.78	
VMELSDLALK	505	2,Ox.[M];	1134.61	
EPQLEDTWLQEPET	506		1714.80	
KDPLLDSLVPAAAAGH	507		1574.85	
EDLSKLEDELYAQ	508		1552.74	
KVLDPEATGF	509		1076.56	
GLTQGEEYMRF	510		1330.61	
KFTNFTLQNFKTF	511		1635.85	
TVMPDVSSKAML	512		1278.64	
KDFVLAEWKQK	513		1391.77	
EPEMVKFEAVEY	514		1470.70	
KKDLDDLELEKDHST	515		1785.89	
KLSCWGSVTETVAK	516		1508.79	
LDDLSSNMEAVAK	517		1392.67	
EYSLKESLPDGQ	518		1365.65	
KDKLDDLELT	519		1189.63	
KYPGLADMR	520		1050.55	
GLGGPTMKQGPLNGPL	521		1536.83	Y
PQDMNKKPGMVTFQ	522		1620.78	
PASALHQDNVDNRSAL	523		1707.84	
LLMPDLVTVK	524		1128.67	
GPAVGPGAVFLRPGYT	525		1558.84	
PPCRTKYETD	526		1209.55	
ESRLDEELSER	527		1362.66	
KEGANKLDGMLPAQK	528		1599.85	
PLSHELTGPGFSWKMAAGH	529		2022.98	
PLGGYQEWDPGHQCSLPEVL	530		2225.02	
FMPGLADMR	531	2,Ox.[M];	1053.49	
GPGGPVGLSYCSALLK	532		1518.80	
LLDVHFENR	533		1142.60	
EVRGPGLPGPQEFVGAP	534		1706.87	Y
LDEALTSKL	535		989.55	
EANATRWYAQWAELE	536		1837.86	
THSLPKFEELLTLR	537		1683.94	
EMVEQENLVTK	538		1319.65	
KLDAQLHLDDAVRGL	539		1663.91	
GPGLGPVQAVDLR	540		1278.72	
STGVFTTLSVAA	541		1153.61	
KGNQLDNLQRVK	542		1412.79	

Sequence	SEQ ID NO	Mod. Sites	Mass	DPPIV motif
ELPVKHGEPLPERGHW	543		1880.97	
DDMEKLWHH	544		1210.53	
YVVEGMRFTTLEAK	545		1643.85	
GLHETTYNSLM	546		1265.58	
GLLNSAGDDGMLGLD	547		1447.67	
EASLKRSTLLSQTHK	548		1698.97	
GPASCDHLVLLEGL	549		1423.72	
GLGKSNESFGPGTLEYGVP	550		1908.95	
PVLDTYVPEMM	551		1294.60	
PLVTLLDGDHDLKRT	552		1692.93	
DPGSLTEEQLAPGLH	553		1563.75	
GEEQVKLW	554		988.51	
PAAETAPLPHWLTQLTPGLAP	555		2181.17	
GQAPKQMLVLQEEVASDPL	556		2053.08	
RVFDKEGDGTVM	557		1353.65	
PAVMTYGSKCFLLNF	558		1690.84	
KEALAGYPDKKVLGM	559		1619.89	
LTDDLQAKMWLEPQ	560	9,Ox.[M];	1703.84	
DESTGSVAKFR	561		1196.60	
GVGGVLTYPSLPLNL	562		1499.86	
PEGGAFMDNK	563		1065.46	
SPPETRLEHDKGHVLTE	564		1945.00	
GLGPFFNKGTWNPMPVLQ	565	14,Ox.[M];	2019.03	
GPFWRTGPSGKLWR	566		1644.89	
GEGATLEDFKMVGT	567	11,Ox.[M];	1470.67	
EVAGTKLGDSEFVTK	568		1580.83	
KLLGDELNKQELSDL	569		1714.91	
KELPEGQFMDGGK	570		1435.69	
WSKKMEKLWHH	571		1509.77	
ATESFGPGTLYPPST	572		1524.73	
HHVVKPNVSVVDL	573		1442.80	
SVRLLYGGS	574		951.53	
LLEKQFSVVDKEKLDKF	575		2066.16	
LQGFDPETAGGFLETF	576		1728.81	
KKNVTRPASYDALKK	577		1718.98	
GLTTWWTPDGLGKKN	578		1673.85	
KGKFGESTTVLPGEMKGKY	579	15,Ox.[M];	2073.09	
NSYKEALDLH	580		1189.59	
VPSGAEQYQQSVNGF	581		1610.74	
NDLLERPGM	582		1044.52	
SPSEGADYYLR	583		1257.58	
KNEEELKTVTN	584		1304.66	

Sequence	SEQ ID NO	Mod. Sites	Mass	DPPIV motif
EVGARAEETLEAT	585		1375.67	
GEGTAGDVAFLK	586		1164.60	
EVLNANQDLLNAKQ	587		1569.84	
KLAPEFVDLLNAK	588		1457.84	
KDESQKYEEELKVL	589		1737.89	
GLGLMMTMLDQAKQ	590	5,Ox.[M];6, Ox.[M];	1568.75	
ATGFKAWDDGWPE	591		1479.65	
VPDSEPKGELLFLESVPD	592		1971.01	
GTAMKLLGCFTGL	593		1311.69	
EVGAGMQSGLDPTQ	594	6,Ox.[M];	1405.62	
ESVLDMLKD	595		1049.52	
ELWVKALVSM	596		1175.64	
KADPTNLHQRVML	597		1522.82	
KDGKNDAPALK	598		1156.63	
FVDLLNAKQ	599		1047.58	
EMDDKLWNWMEKLEGH	600		2060.91	
PAESEKNLLFDK	601		1390.72	
VELQQDDLERTETVPVMMRAP	602	17,Ox.[M]; 18,Ox.[M];	2489.22	
WNDDMEKLWHH	603		1510.65	
EEPFHLR	604		927.47	
ENCVLSNFFSH	605		1296.57	
KEDLLVDHEMMKFGHTK	606	10,Ox.[M];	2074.02	
KDGYLPGFSGL	607		1153.59	
KDPLLLGMD	608		1001.53	
KGSPGLFGDPWRNGKVSL	609		1915.01	
TVYPKYDLMKYPTVLTWAEV	610		2417.26	
KVKNEKAPDHWLKKVVAK	611		2118.25	
KGVLGLPEVYGTVQAP	612		1627.91	
PVDSSMNKKHTTFQ	613		1619.79	
TVPRPNVSVETQVVNM	614		1769.92	
ELPMCVELYLK	615		1337.68	
GFAGPPGGDQGPGAK	616		1312.63	
GPVMVGHELEEMQ	617		1455.66	
THSLPKFEELLTR	618		1570.86	
KSLVEDEQSLQQLQ	619		1644.84	
LVEPQKEPGVAPF	620		1410.77	
KAAEEQFMEGLAQAKAE	621		1850.90	
YPGLADMR	622		922.45	
KELPGPQLLEKAVP	623		1518.89	Y
EGAPGFMDLFAATPQ	624		1551.71	

Sequence	SEQ ID NO	Mod. Sites	Mass	DPPIV motif
QDAGALWLTH	625		1111.54	
GQRFTTLSVAA	626		1150.61	
APAPEPPEAAVVAL	627		1331.72	
GLDGLNKQKMSLPTSGL	628		1758.95	
KGDVHFMDLLGKDVFH	629		1857.92	
ALGSWELALEEAKKKLANVLA	630		2254.29	
MYPGLADMR	631	1,Ox.[M];	1069.48	
TLAWPYYMMLPTEK	632	8,Ox.[M];	1759.84	
VDASRLTPSL	633		1058.58	
KDLLDLAGDDPVPLLGWVLAH	634		2257.23	
KAWNKPGDGKAAQEEMLKVK	635	16,Ox.[M];	2244.19	
PAAALFVLKNLPVDF	636		1614.92	
LTDKVLENR	637		1087.61	
KAYEEALDLH	638		1188.59	
FAEDPLFAK	639		1037.53	
LGSVTESLK	640		933.53	
LGDAETVKGYRPG	641		1362.70	
GPVTFGPELQWQVTGPF	642		1859.93	
PELDGKLTMYLDGKLT	643		1793.92	
AAPQQDMADVGF	644		1249.55	
EVASKDYVEGDPL	645		1421.67	
PVMHNRPGPLGPSHQKR	646	3,Ox.[M];	1923.99	Y
PQGLEVLVPHPL	647		1298.75	
PAAPPEDPVAAKQAPQNYN	648		1977.98	
EVPGSPEVELVEKY	649		1574.79	
RELEELSERL	650		1273.68	
KAPMKENRLCHEVKAK	651		1882.03	
SGGFRLLDPQPMPSKSF	652		1863.95	
TVALFHKPAALGSPWVF	653		1841.01	
DELASWDGDELAYLK	654		1724.78	
KLGEELLYLK	655		1205.72	
LGFAGDDARPLGFSP	656		1519.75	
KPMMVPGEQAVFPPLMAK	657		1971.01	
LLTMYWNVPPF	658		1380.69	
GPQKGYPSTPLTPFP	659		1586.82	
KAFVSVNPHVAK	660		1296.74	
HEWMKTL	661		944.47	
AAVDLAPVSEK	662		1099.61	
GLGYLLEENQPFTVP	663		1676.84	
KFVQRHEPDLTKH	664		1634.87	
NSGWGVMWVYGPM	665	7,Ox.[M];	1499.64	
KGLNEPLVTL	666		1083.64	

Sequence	SEQ ID NO	Mod. Sites	Mass	DPPIV motif
DSPQMMGELGKLNVW	667	5,Ox.[M];	1720.80	
KEGKLMGDLAVSKEDRVEPDK	668		2344.23	
PDTEYKVLVT	669		1164.61	
SVALSPDQPMSVLEKAL	670	10,Ox.[M];	1800.93	
GPVAGDLGFMWVLTL	671	10,Ox.[M];	1591.82	
LTPWGQKELCVLAHK	672		1722.94	
KAAALGTVTQGPL	673		1226.70	Y
VDQEKLDHF	674		1130.54	
SPGMLKPSSL	675		1016.55	
KVLQEVLQVKLW	676		1482.90	
KAQKLPSVELT	677		1213.72	
DFHEAMRVRDVY	678		1537.71	
KDPLLLGTNGPQLLGM	679	16,Ox.[M];	1682.91	
KSVEENESLGAQL	680		1403.70	
VPDLSMAVWAKTLVQNAL	681	6,Ox.[M];	1972.07	
PVHEQDALLDDAVR	682		1577.81	
TTTRSNLDAMFEAY	683		1619.74	
RQLYEEELRE	684		1364.68	
KPLTGYLKRLLLETPNP	685		1953.16	
KATSMKFMEGSLEFVAVP	686	5,Ox.[M];	1988.00	
AAVPSQENVPNLQ	687		1366.70	
VTDLFKFTYWMMGFLGT	688	11,Ox.[M];	2072.99	
DPLGELLPPTVLPD	689		1475.80	
EGGKVKLEGELM	690		1289.68	
KPSDLPAVPWPAST	691		1465.76	
GVGSGNVAELK	692		1030.56	
EFHRKKCHFELLAL	693		1770.94	
GPAGDVLSRMMVTL	694	10,Ox.[M]; 11,Ox.[M];	1478.74	
KAQQGHLPNEAEDHQGPVVQ	695		2182.06	
KMNEFHPVGFASK	696	2,Ox.[M];	1507.73	
GPATKVLPYYLLGPF	697		1635.92	
KAMKMMVMDTFLADL	698	3,Ox.[M];6, Ox.[M];	1776.85	
ALGGNQPVTNGQLLL	699		1494.83	
PDGQVLPVGSSHGVHVTLH	700		1935.99	
SEELALDNAPCCYAL	701		1611.70	
AEGTKNHEESKLL	702		1455.73	
DEAGMAGPSLVHKR	703	5,Ox.[M];	1483.74	
SEEMLLNEF	704		1111.50	
LAEEADRKYEEVARKL	705		1920.01	
GFAGDDAPRAL	706		1089.53	

Sequence	SEQ ID NO	Mod. Sites	Mass	DPPIV motif
DHFVNKKTFVKL	707		1475.85	
KNPDGTFDDYQTGL	708		1570.71	
KAAAKVSPDMMELRPLNL	709	11,Ox.[M];	2000.07	
GPDCPLEHEESKLL	710		1566.76	
KGPVMLVQVDTVRGL	711	5,Ox.[M];	1627.91	
NSYEETVDLH	712		1206.52	
GPGKVALEEEADE	713		1343.63	
NFHEAPAVENFVY	714		1536.73	
DNYHLLEGL	715		1073.53	
SQKEDKYEEELKVL	716		1737.89	
KGPEVDEVATVK	717		1271.69	
GTEFMQDDPVKGGH	718		1517.66	
DMEKLWHH	719		1095.51	
EVDFKSPDDKQSWSRT	720		1924.90	
YPGLADMR	721		922.45	
LAGVENVAELK	722		1142.64	
KHLAVPMTMLAHKLLGTLQ	723	7,Ox.[M];9, Ox.[M];	2134.19	
KELLMDGLYER	724	5,Ox.[M];	1382.71	
KEVTSQGLSEKLEMT	725		1679.86	
GPAGSGPEPPGPNKSGPQGF	726		1834.87	
VPGVKKREDELDYK	727		1675.89	
GTMDFRAN	728		911.41	
LVEEELDAR	729		1073.54	
KVVWLLADPVL	730		1252.76	
PVDGLVMLLDTVR	731	7,Ox.[M];	1443.77	
KNLPCEELNKLL	732		1413.77	
KGVVLSTEGPTAPDFPNKPT	733		2055.09	
KPVHRLQETSQERFQVPL	734		2192.20	
KMLDHEELYDWEPTP	735		1902.87	
KAELQNMVQPVL	736		1369.76	
KGFTVYLHNVLQKLEGNH	737		2097.12	
GLPLVEVDAK	738		1040.60	
TVTDEALMPR	739		1132.57	
PVADGHSVLWTF	740		1328.66	
FLLKLDHF	741		1032.60	
LSRELKAASYPWMK	742		1679.90	
LVRSEFAELVEMHQQ	743		1815.89	
LFDKPVSPL	744		1015.58	
GLGYLLSVESVPPSFL	745		1677.91	
KTKDESLGKVLENR	746		1616.89	
GLSEQEYEFR	747		1257.57	

Sequence	SEQ ID NO	Mod. Sites	Mass	DPPIV motif
AAFPPDVASQADFK	748		1463.71	
GHAGFVGLPASTSRRQPGGPG	749		2006.02	
KNFDYEDELNKR	750		1570.75	
KAADYLEELQNGPK	751		1575.80	
PVHAASAGPHLDDAVAR	752		1683.86	
LEGSLELVALPQ	753		1268.71	
KLGVEDELDYK	754		1308.67	
APGWMVMTVNTLLQ	755	7,Ox.[M];	1576.77	
TLAWPYYMMLPTEK	756	8,Ox.[M];	1759.83	
KGVAVLENPLGK	757		1224.73	
AFRVPTPNVS	758		1087.59	
GEELFKMK	759		981.50	
PASKELYMASYDALKK	760		1814.95	
GTNLPEVVWSK	761		1229.65	
VDWTAHLFLGME	762		1418.68	
GPTLGPYSFGPGSK	763		1364.69	
KDGHDKLQMKVK	764		1426.79	
KQSEHDLLDTVR	765		1440.73	
SVRLLYGGSVT	766		1151.64	
ELGATYMPMLKQKMGAM	767		1899.93	
DLDLRKDLYAN	768		1335.69	
PVRTPNVSCLVTNVT	769		1599.87	
LVQEWGVLAL	770		1127.64	
KNKEADPYPGQLSLEDQLW	771		2231.12	
VEATDLEEK	772		1033.50	
GFTNDEKMAL	773		1125.52	
EPLGGFYEQLEHL	774		1531.75	
KFLLEEFKLCW	775		1455.77	
KDVAVELY	776		936.50	
KVNGTPLDTYKMDLTFKAL	777	12,Ox.[M];	2171.14	
VVGDVETVTNPK	778		1257.67	
GSELVFTAK	779		951.52	
RELEELSERL	780		1273.67	
GLGGKGEQLDNLQR	781		1484.77	
LSPSFVGTWKM	782		1252.64	
KGLNEPLVTL	783		1083.64	
HNLDDELLK	784		1096.56	
DEFLDFLKMM	785		1288.59	
VHDLKPDNFLRL	786		1466.81	
LEDFQSLFGL	787		1168.59	
EALRNNQLAEAE	788		1357.67	
GVAYGPVVMSWLTDK	789	9,Ox.[M];	1638.81	

Sequence	SEQ ID NO	Mod. Sites	Mass	DPPIV motif
DSYEEALDLH	790		1191.52	
EPDVLFVTNPKRLQ	791		1655.91	
APLGPLSEYLAPAPV	792		1494.82	Y
PLRLPVGPETL	793		1191.71	
KVAESGPLNF	794		1061.56	Y
EQLQVDDLETREQEQKLSSL	795		2388.21	
GVAAPQPVVGGVAYMDVGGL	796	15,Ox.[M];	1872.95	
KLNQSNEELKAR	797		1429.78	
ELGFGMSLVQLLWLAM	798	6,Ox.[M];16,Ox.[M];	1839.95	
GEGAAEVVGLK	799		1029.55	
KDALAMAASEVLVGPD	800	6,Ox.[M];	1602.81	
LEEMMGNAVKKKM	801		1508.77	
LVSTWGQELGAK	802		1288.69	
ELTMFHAPDFHRPF	803		1744.82	
DPELMPQLPNMDALCKTVP	804	5,Ox.[M];	2128.02	
ESPGLSFQLALAAPA	805		1471.77	
KVLDNLLDVFGPA	806		1400.77	
PAGHMPGPVQNY	807	5,Ox.[M];	1283.58	
KEEALELVS	808		1017.55	
KLSSMEKLWHH	809		1395.73	
KMAGPAHFQAGKL	810	2,Ox.[M];	1371.72	
KGCVYDNEFGYSNR	811		1651.72	
KMPGLGLAEHTSKLGY	812		1701.90	
LGRPEEHPTLL	813		1261.69	
KSLEEGYRTFEESKLL	814		1928.99	
KGTALLTGELYVDSAAN	815		1722.88	
LAQRTEELEAE	816		1288.64	
TFGLYFLYLMK	817		1395.73	
VPAHFEYELGPD	818		1373.64	
ALAGLKGDLAGHYY	819		1448.76	
APGGSGLLLLPANGSLDGY	820		1771.91	
VPDEVKLTDCGLT	821		1389.71	
VVEVSVNPHKGL	822		1277.72	
GWVGVEMWGVVGLK	823		1516.80	
QVPMCHPFHLR	824		1364.67	
ALPHNKGLMVGAL	825		1320.75	
ALAKDLLEKE	826		1129.65	
WAGLEDFVPAPG	827		1258.61	
KFRFLAEDPAAF	828		1411.75	
LHVMTPMYEDPRMVEGL	829	4,Ox.[M];	2033.96	
LLAEVELK	830		914.55	

Sequence	SEQ ID NO	Mod. Sites	Mass	DPPIV motif
VLGCLALELLK	831		1171.71	
GEAEVELK	832		874.45	
VPHTELTGPGFSGAVPKKYDAPV	833		2367.21	
LAMYPGLWCAL	834		1237.60	
KEDLLDTRVSL	835		1288.71	
PVELLDMSLLEFGPV	836		1658.88	
KAEVLMFSVGVVP	837		1375.77	
GVGAKELWLTL	838		1186.68	
EGVDDLVQEAR	839		1230.60	
EVAALFGSLLTT	840		1221.67	
ELWVELGDKLGFAGK	841		1661.89	
LTAAEELELSELR	842		1473.79	
PVEELRWDFKGL	843		1488.77	
KDLRHKVPGLTF	844		1410.82	
GEPMDLVMLLAN	845		1302.64	
KDTVEVPVLEQPV	846		1452.80	
ELEKVMSQMESLEEELKT	847		2153.06	
VPGDALKPHCVLPNAL	848		1643.89	
PEATGLWVVTWPKATGF	849		1859.97	
EHRDPANLKWGDEAT	850		1738.83	
TLRYEVMSPGTLPHSRPA	851		2012.04	
GFTNEEKGL	852		994.48	
KDGLLWQPVEAGL	853		1425.77	
KEYEPEMGKVF	854		1356.65	
VPGSDVPELVEKWPPET	855		1878.96	
LVDASERVGL	856		1058.58	
ATGGPFGVPTKMKVTSF	857	12,Ox.[M];	1740.91	
LETTLPLRPLK	858		1280.78	
KFGGTDGYETVMNPK	859		1643.77	
KDPLLYRKTWNLLGM	860		1848.03	
EDAFEEEVKAK	861		1294.61	
GLGPTADLVGLR	862		1168.66	
DEELLVSGFLEWKLSRCT	863		2125.07	
PTGLNYVLHLPCP	864		1423.74	
GLGVRLLNEAAPT	865		1310.74	
DPKEELDRAQELR	866		1598.83	
KDLVDLDLLNPL	867		1367.78	
DDMEGNEKLWHH	868		1510.65	
TVAFGRFVAEAP	869		1264.66	
EVSSNMEAVAK	870		1164.55	
KGVAVLPELDGK	871		1225.71	
KVEASGPLLMALFAF	872		1593.88	Y

Sequence	SEQ ID NO	Mod. Sites	Mass	DPPIV motif
EVQHRLDEAENL	873		1452.71	
GPSGDRQESGLQTL	874		1444.70	
GLFKWDGLYER	875		1383.70	
DHLAELTLFEEYLMTDMTL	876	17,Ox.[M];	2301.09	
KWQEAKYEWQAELEGAQQ	877		2222.07	
GTLLVLGVPV	878		967.61	
FVAVKDVVDEKFLGM	879		1696.88	
LTDEALMPR	880		1045.53	
KGKPEFVDLLNAK	881		1458.84	
GPGGHGTLEYRVP	882		1339.68	
LAPYDTPLPGYVADV	883		1590.79	
KPGRGPGMTSPATWGGLPV	884	8,Ox.[M];	1881.96	
ALEEELRGF	885		1063.54	
KGVEPELVGKTL	886		1269.74	
LHVDPDGVRAF	887		1225.63	
KMLLPQAGPNPVMGLQKWPVVQ	888	2,Ox.[M];13,Ox.[M];	2463.31	
AFPPDKDVGNAVYQ	889		1520.74	
GPGDNFYLFEGK	890		1343.63	
KAELRHSSCFLHLK	891		1668.91	
LQVLRRPVTVF	892		1327.81	
LVDMLDLMFYALK	893	4,Ox.[M];	1587.83	
PGALDGVMQKELYDV	894		1634.80	
KGVVLPELSNPYGAL	895		1556.87	
DHLALETL	896		911.49	
AFPDVDFLKN	897		1165.58	
SEFGPGTLMSTPL	898	9,Ox.[M];	1352.65	
HDYNHLVLLEG	899		1309.65	
DGAYEVVVLKL	900		1205.68	
KPDLLPLLQP	901		1133.70	
GELWHSDLAHK	902		1292.63	
LLKSNHELKGLR	903		1407.84	
RADLSRELEELSERL	904		1815.96	
FNVLDNSHAGNLK	905		1428.72	
PAGGSWGKLNFLTGPA	906		1572.82	
SHCSSHLEMR	907		1186.52	
SPADAVMEWWAP	908	7,Ox.[M];	1375.60	
DPEVKPVLELK	909		1266.73	
KDMPFYNCHGSML	910		1542.64	
PLQDVYLLVCQQKGL	911		1716.94	
LTENGFEVNLQ	912		1263.63	
GLETELVDASEKSP	913		1474.74	

Sequence	SEQ ID NO	Mod. Sites	Mass	DPPIV motif
VEDSMGQPVQVR	914		1344.66	
TLARAPDFSYGRAL	915		1537.82	
EVGYKSLPELGDLPMPAL	916	15,Ox.[M];	1944.99	
ELRLGLEQ	917		957.54	
TLGMTLCELGTM	918		1269.58	
ELTMPVEGLVVLQ	919		1427.78	
DPGQVLNRDCLPDNVGALLT	920		2110.05	
DHDFVSTFVKL	921		1307.65	
ESRLEELSVWHSAF	922		1689.83	
KEHLELR	923		924.52	
VPSNLSGKTEGLFEVGH	924		1770.90	
FTPENQFFDEAATT	925		1617.71	
SVGLQGHADQPNPPGYFL	926		1896.93	
KEVKHVPGLTFL	927		1367.80	
NPPALRYVSVVD	928		1329.72	
KPMMVPGELLAQ	929		1313.69	
DPAGATKPDVLAK	930		1282.71	
GLGELRLNQLK	931		1240.74	
KDPLLLEPECVLLGM	932	15,Ox.[M];	1685.88	
VPSTTNLHNDEKLGL	933		1637.85	
LLEWEESLK	934		1146.60	
TLTTMDTTLEAK	935		1324.68	
AHDLELRHLDHAL	936		1539.81	
PGGETKTLLGPAM	937		1271.66	
KAANELEELSERL	938		1501.79	
SHLVDGFDVLWSH	939		1511.72	
SDHEMHFPDLSLH	940		1564.68	
KDWSELVELK	941		1246.66	
KSQLEDDLGL	942		1117.57	
KGQQPGLPGPGC	943		1138.56	Y
KEGKLGMDQTLTANLQKEVNQ	944		2345.23	
EGQLGNNKDEEELNAE	945		1788.77	
GPLADRMQKELTDFT	946		1721.85	Y
THSLPKFEELLTR	947		1570.86	
EDLDDELTKRSYAQ	948		1682.78	
LLTANDDDMEKLEMAL	949	14,Ox.[M];	1837.87	
HLLDDAVR	950		938.51	
LSDLALK	951		759.46	
TAGKKWKLNAPDRMT	952	14,Ox.[M];	1732.93	
LTFSYGR	953		843.44	
DPGAPEDQELVCLALK	954		1697.85	
LVVTNLELDNPL	955		1339.75	

Sequence	SEQ ID NO	Mod. Sites	Mass	DPPIV motif
KNPVALCALTAACKVALQ	956		1814.02	
WHLKARQLWHTH	957		1612.85	
GPAEYEEELKVL	958		1376.69	Y
NWGALTRGPVQN	959		1312.69	
PEGGAFMDNK	960		1065.47	
KDAVLLVAFAEAAH	961		1454.81	
KDTVELVPWAQLPYLY	962		1935.02	
KAMEEEEVKAK	963		1291.65	
LPYTGHAPLYEGY	964		1480.70	
GPLTEDGGFTKEPA	965		1418.69	Y
EASDMMQLKFM	966		1330.58	
GPLTVFWLPQLATGGPLL	967		1880.07	Y
DGLTLGFASK	968		1008.53	
KPPDYTYYGLEDM	969		1591.70	
KDGVVLGAAKAGPV	970		1281.75	
EYLTAYPLYEGY	971		1481.69	
SEVLDMLSRAMKG	972		1436.72	
KTRTDDLHLLMLLCT	973	11,Ox.[M];	1788.95	
EGERSELEGEESRLT	974		1720.78	
KDGAVTGELLK	975		1130.64	
KFDSVEPADPDSQYVPMKL	976		2166.03	
LLVRLPGEDAKAH	977		1418.82	
LGMALLEELSEPTSL	978		1602.83	
NHGDGAQEAVRL	979		1266.61	
LTGPLPKQPLVY	980		1325.78	Y
LEPVKHTGTYKVELEPYEL	981		2245.19	
NLLFLANMK	982		1063.59	
VPGDLTAYQATLPAPG	983		1570.80	
TVEVMEVTVK	984	5,Ox.[M];	1150.59	
NDALNYKCLPDD	985		1380.60	
KNQQLSDLQKEQQNQRWEPA	986		2468.25	
EASLGLPMLLLT	987	8,Ox.[M];	1273.71	
PLLKAGTLAQAMSWGLGKAQ	988		2041.15	
GVAYGPVVMSWLTDK	989	9,Ox.[M];	1638.81	
SESLSRAQEKLEGL	990		1546.80	
KLDAQLHLDDGLR	991		1493.81	
ALDTELAVGGL	992		1058.58	
EGFELMKAVAHL	993		1344.71	
KGDLVPVLGKDVHF	994		1523.85	
KDLRKHVPGVT	995		1249.74	
LLENVDRF	996		1005.54	
GFGLVDLGWGPVGAH	997		1481.75	

Sequence	SEQ ID NO	Mod. Sites	Mass	DPPIV motif
TPTLPMLWMKSPELPPLAH	998	9,Ox.[M];	2175.14	
TVHPWLNKYEDEVQKR	999		2042.05	
WALRKWYDDVWAF	1000		1755.86	
EDTGEFVLKPA	1001		1205.60	
DFVAGRVVGKL	1002		1160.68	
KELHCAHKGLVLEAKHCL	1003		2029.09	
NSGLSVWLEEAGQ	1004		1389.65	
KGWVDLGPHLGPLN	1005		1502.81	Y
DELESELR	1006		990.47	
HELFTRMLEMH	1007		1443.69	
PTNDAFAKLCVE	1008		1307.63	
LVPEGQFMDNK	1009	8,Ox.[M];	1293.62	
KFVQMEYPDLTK	1010		1498.75	
ALGELWECASLAPA	1011		1430.71	
SFDTPPPFMCLDAHFKH	1012		1989.89	
PAGAVHEWTQLVKSH	1013		1659.86	
KGMSVGDVNWQPASAL	1014		1659.82	
VVMVSVNPAALGLDL	1015	3,Ox.[M];	1513.84	
KNSGGYSFVTTA	1016		1231.60	
GLDAPPEAEDMVLN	1017		1470.68	
TVEEMLGWFKAA	1018		1381.67	
EYDLEGGKQVFM	1019		1415.65	
LVPELDGKLT	1020		1084.63	
PPCRGNDETKMLLDPD	1021	11,Ox.[M];	1816.83	
KGVSLMQPHNVF	1022		1356.70	
GPVNVPVGPLEFK	1023		1352.77	Y
MNSHNVRADFPYLR	1024		1719.84	
QSEKLWAFFNTH	1025		1507.74	
KSTPLWLLTGE	1026		1244.68	
EHLGETEYLF	1027		1237.57	
LGEGSLALEYSAHLQGGH	1028		1838.90	
GPGPQLHRELTAL	1029		1388.76	
EAGAGYTMVQVLKFGHVQ	1030	8,Ox.[M];	1950.99	
GLGKLEEDWWELQGGLL	1031		1943.00	
LAGFGELHLSRMLGLR	1032		1770.00	
LHLGLHEWMK	1033		1263.67	
KDPLLGYVCHNVLGM	1034		1658.85	
EGSLRMSMLGYNQ	1035	6,Ox.[M];	1501.67	
TTGLVMPSVGVQ	1036	6,Ox.[M];	1204.62	
WHLKPKKLWHHT	1037		1610.92	
PEEHPTNRPTLL	1038		1403.72	
KSTLALDGQVLGQ	1039		1329.74	

Sequence	SEQ ID NO	Mod. Sites	Mass	DPPIV motif
EVGSEYMPDLPNLQ	1040		1591.74	
GNSGLDSLLCPGQ	1041		1260.59	
APGGCAQEFVRAL	1042		1318.67	
YMVPSNENELLKAPQ	1043		1732.84	
SNLALCVNTV	1044		1033.54	
NPPGEALTTPCPMHKNTL	1045		1920.94	
LMLDVEAR	1046		946.50	
KFPEEMVEYK	1047		1299.63	
KDVLTSVPVEEM	1048		1346.68	
VNDLFYQPAVP	1049		1262.64	
KAGAVCKKLGAKAK	1050		1372.85	
KGASVLGMDLCVKWNTSPDAKK	1051		2348.20	
LEESLGRL	1052		916.51	
EAGDKVNLTVVQGPL	1053		1539.85	Y
AHDLFPWRREFHADLAL	1054		2094.06	
LVDASERVGL	1055		1058.59	
KKDLDDLTLPQLTDDSH	1056		1953.99	
VVGDDLTVTNPKR	1057		1413.77	
KAAAFSNFFSH	1058		1226.60	
EGRGMLQEEVAGQAR	1059		1630.80	
EAPLQADNGVPVMAQ	1060		1539.74	
KEGVETTTKEPTPQ	1061		1544.78	
KDVLDPMDVDLK	1062	7,Ox.[M];	1403.70	
QSYFKENKDELLE	1063		1642.79	
KGVEPEPDGKLT	1064		1269.68	
GHMGVSPSTVF	1065		1118.53	
LVFPWTRQH	1066		1183.64	
ELPALMAPGPGPSF	1067		1383.69	
PVDFEEDFSFLK	1068		1472.69	
KNAQDEELNKLL	1069		1414.75	
ERELKAVSRWVVP	1070		1568.88	
LVGPHEALQPTSATGQLQ	1071		1846.98	
TVPRPNVSVVD	1072		1182.65	
ENEELSERL	1073		1118.53	
LLRYQRVCHEFLKR	1074		1861.04	
PVAPETLENVGFWGVM	1075		1745.86	
KSLVEDEQSNKLQ	1076		1517.78	
SVLKVGQFMDNK	1077		1365.72	
DELKPVETHATWLDDGLR	1078		2095.05	
GFVGLATGGPDSKGLPGGPG	1079		1740.90	
NHGDGAQEAVRL	1080		1266.61	
GEVDDLLWTTLVKEAR	1081		1844.96	

Sequence	SEQ ID NO	Mod. Sites	Mass	DPPIV motif
PASAQMSRMQLPASAL	1082		1658.83	
TLDYLPMNLMPAL	1083	10,Ox.[M];	1507.77	
KTDTVLVRQPNTL	1084		1484.84	
ESRLLEEPTELR	1085		1471.78	
KNATVQLVPHPL	1086		1316.76	
DDMEKLWHH	1087		1210.53	
PHESVEFLPV	1088		1153.58	
EMNPKVMEKLEDM	1089	7,Ox.[M];	1609.72	
LHVDPDLTGKPF	1090		1338.71	
KDNLPGGPGAVGAANPPEVV	1091		1858.98	
DVETERTEAADKKLVSDT	1092		2006.99	
LGDTSALWVGF	1093		1165.58	
KDESQKYEEELKVL	1094		1737.89	
KGSSQPKWMKGAVMANFTLEGL	1095	14,Ox.[M];	2396.22	
LLGLVMPLSTPL	1096		1253.74	
GLETELVDACLGV	1097		1318.64	
ALTPALYGKL	1098		1046.63	
TLEDGLFEVK	1099		1150.60	
TVPRPNVSVVD	1100		1182.64	
SEEDTLYYKKM	1101		1406.65	
GPANMSGLGPPLCADFLLK	1102		1900.98	
GLGQHPPSVLSMKL	1103	12,Ox.[M];	1479.81	
SEALLPKLGL	1104		1040.64	
LTDVHTPF	1105		929.48	
ALGELWECASLAPA	1106		1430.71	
KGEQDERSVLLT	1107		1374.72	
CHPFHLR	1108		909.46	
GLASTMEAAAPGLHMLPGY	1109		1886.93	
SESLKEGQELK	1110		1247.65	
STDDTFLLEPLKGTQLGE	1111		1963.98	
PLPELNGK	1112		867.50	
ELEEELEKLLEQNK	1113		1743.89	
TVHTLFDGELGCK	1114		1419.69	
TVLEPRPTEAF	1115		1259.66	
KPMYLALGPLTAFGKLK	1116		1848.06	Y
LGGAYLNLFLAA	1117		1222.68	
ELLREELGDK	1118		1201.64	
LDDAVRGL	1119		858.47	
TVAFVNKCTAPL	1120		1263.67	
FAKLEPWMAAKAQ	1121	8,Ox.[M];	1506.78	
KAVSTYSLCFKWELFAF	1122		2040.04	
EGLAPETEVTCMML	1123		1523.67	

Sequence	SEQ ID NO	Mod. Sites	Mass	DPPIV motif
LLHAVKDLFNLMCPL	1124		1726.94	
EVEEELTPFLPD	1125		1417.68	
EVVLEKCWLAGP	1126		1343.69	
STELFKKL	1127		965.57	
GPADLMLDLGPA	1128		1169.59	
KGVLGLPEVWKLT	1129		1439.87	
LPDGPTRDFLPLVT	1130		1540.82	
KVLGPGVTLQAPQL	1131		1420.85	
FPPDVPDKYLM	1132		1321.64	
KEGWDLPAKQ	1133		1171.62	
KYAQGLYKLTNW	1134		1484.79	
TVDLATRKMAPTAL	1135		1487.81	
LGADELTKSRLDWMDVLPL	1136	14,Ox.[M];	2188.14	
PVGGFSGMLGQLF	1137		1309.66	
GEGGTMKMKGEFGRQL	1138		1725.84	
EVEDALQAKR	1139		1158.61	
GPLAFSRGKPLGPY	1140		1459.80	Y
EEELEKVMTMLSPV	1141		1634.80	
EYSLKELMNPGLPT	1142		1591.82	
KGSGLDRLLKTM	1143		1318.75	
KETQESHDLLEKE	1144		1585.76	
EMVDFTETLTPLQ	1145		1523.73	
KACPAGDVTYNLT	1146		1352.65	
EEDHLRVL	1147		1010.52	
SPLVGEMDNPCLPG	1148		1428.65	
KFPYLVPMDSVKGQ	1149		1608.84	
EPGQFLDTVNM	1150		1250.57	
EYNELAMRQ	1151		1153.53	
ALGAWVDTALLPMLVP	1152		1666.94	
DLDLRKDLYAN	1153		1335.69	
KGEVPVGEVCESKVM	1154		1590.77	
GPGAGPVYLGVTTHVVQGAP	1155		1876.98	
AHEELKAR	1156		953.52	
SVETELELMGPAL	1157		1388.69	
KNPDLEESFNLSM	1158	13,Ox.[M];	1539.71	
KDKLDDLELT	1159		1189.63	
GLLETESLGEPLTAL	1160		1542.83	
TPGLNLFLMAP	1161	9,Ox.[M];	1189.62	
KGGEADDNMLGLD	1162		1334.59	
PVDFGTVMESFLK	1163		1469.73	
EYFDQTKFLYSQ	1164		1568.73	
EVEDALQR	1165		959.48	

Sequence	SEQ ID NO	Mod. Sites	Mass	DPPIV motif
TVLEEEAKFTK	1166		1294.69	
TFYDQLR	1167		942.47	
ELSDLNRMLQ	1168		1218.61	
GENEAKLLTL	1169		1087.60	
STTYSLLEKYVGRT	1170		1617.85	
KDESQKYEEELKLL	1171		1751.91	
KALTELLECMLAAP	1172	10,Ox.[M];	1518.80	
VDDEKVVWDEKVVAK	1173		1758.93	
KDVLLSGTDVDLK	1174		1402.77	
EGALGKLLWQKQLGEQ	1175		1797.98	
EHLGYGLLGYTE	1176		1351.65	
GLDGLGWGLDGL	1177		1172.60	
KAELAKLGSL	1178		1029.63	
RDNYQMLLML	1179		1296.64	
TVPHSQLEVNMPLGH	1180		1658.84	
KTWDLVQEAR	1181		1245.67	
EPDVDAKGLQAP	1182		1239.62	
VDEEKLEFGGPA	1183		1290.62	
FLGVTRWLKKKM	1184		1506.89	
NVNAKHVLTTL	1185		1209.70	
GLGGPMVVGLK	1186		1027.60	
LVPELDGKLT	1187		1084.63	
FLGTFLEMH	1188		1094.54	
SVPRFAWSSSEVSAYYVQG	1189		2120.00	
PELMKMTQLMF	1190		1368.68	
KCSFGPGTLEYR	1191		1357.65	
KGPDLSKGTTAGY	1192		1294.66	
EVKRLDDLYAL	1193		1334.72	
GTGLDWVQEAR	1194		1231.61	
ESRLCHLQVELSERL	1195		1811.93	
ESLFRWCSVLENR	1196		1638.80	
LVFRVQYDFH	1197		1323.68	
GEGAGLWMLFYN	1198	8,Ox.[M];	1373.61	
SERLLEEPCER	1199		1360.67	
KGEGDGKEVNLANPGGGVYT	1200		1961.95	
LLPSPLEGDLK	1201		1181.67	
KAKPAVSEK	1202		957.57	
LADRLPSVGEVLWLH	1203		1704.96	
KACPMKELTAL	1204		1204.63	
ERTLEDEEEMNAE	1205		1594.65	
LGGVDVCMKKFVYF	1206	8,Ox.[M];	1621.80	
KELDNGKDVAGL	1207		1258.66	

Sequence	SEQ ID NO	Mod. Sites	Mass	DPPIV motif
KNDFNFVGKDLGFVK	1208		1727.93	
GPGPQTAYDSLM	1209		1236.56	
GLARPLEDQYYYLK	1210		1728.89	
TVSEWYQADLVRAT	1211		1638.80	
RADLSREL	1212		959.53	
ELCWLPVGGRGY	1213		1349.66	
KHCYEEKLATLVGM	1214		1621.79	
ESMYFAPAKQ	1215		1171.54	
LAAPQWHLWHTH	1216		1496.75	
DVAGRNVETMGSL	1217		1348.65	
LHGLDGFMKKSLAVMHDGL	1218	8,Ox.[M];	2085.05	
SPGAVGVGSTVSTLQHEASR	1219		1939.98	
EVFPLDNCNVK	1220		1277.63	
KGTFLEMH	1221		962.49	
TALEEAELPGEALFRLCH	1222		1998.99	
KSAALSKDDTPLHFMVVKGGPA	1223		2269.21	
KDVQSYNQVEYMKAL	1224		1815.90	
ALCCEELQGNKML	1225		1451.68	
GLLSEEEMMFR	1226	8,Ox.[M];	1357.62	
PGELAKHATQLAKAH	1227		1571.88	
GEESRMKVLDAEN	1228		1477.69	
DNWVLATTLGRKGRVPPA	1229		1951.10	
GPPMGPCHQSMPGPMGPM	1230		1808.74	
KPLKGMFELAKVPGL	1231		1627.95	
ENLRFHGL	1232		985.52	
RVPTPNVSAQ	1233		1068.58	
LLTPWDLVDLK	1234		1312.76	
KPTMNQQFSEALNSYKTY	1235		2150.04	
KLVEWMKEVSTY	1236	6,Ox.[M];	1528.77	
ELSSDLVRAT	1237		1090.58	
KPEKFDLHLKMLLGPMF	1238	11,Ox.[M]; 16,Ox.[M];	2076.12	
GPAGPGALASTDGMTPNNPL	1239	14,Ox.[M];	1853.86	
EANQCNLPNVEGL	1240		1400.64	
SSEALKKKM	1241		1021.57	
SVAELLCELGELLK	1242		1516.82	
PVRHVATDLYWLTSLK	1243		1899.03	
GPPNDRNPGPLGPLETHR	1244		1923.97	Y
GPTTSLTTKGQKNFAPQ	1245		1775.92	
KSPCTPYMLGY	1246	8,Ox.[M];	1275.57	
PAMYVNVALQ	1247		1105.57	
KDPLLLEGAPA	1248		1123.64	

Sequence	SEQ ID NO	Mod. Sites	Mass	DPPIV motif
GDDVSKTVTNPKR	1249		1416.73	
GVLEEMRDE	1250		1077.49	
KVLQEGFSMKLW	1251	9,Ox.[M];	1481.78	
ALEVVEEK	1252		916.49	
EVELKLFLQ	1253		1118.65	
GESTAGRLEKSTVAH	1254		1542.78	
AHPLTTVYGYLK	1255		1362.74	
FPPDVKTCSLGGT	1256		1321.65	
KVLDPEKGPDLVGVAT	1257		1637.90	
AAGPMKFDPAKENMK	1258	14,Ox.[M];	1650.80	
TEAVLPYDKVVAGH	1259		1498.80	
KEGTGDKLALLDAKQ	1260		1586.88	
VVPSNVDLTVR	1261		1198.67	
KGAPVEERGLTTY	1262		1420.73	
PAGAEFKCGVAQGPA	1263		1402.67	
ALGELTMEVLH	1264	7,Ox.[M];	1228.62	
SPTKYPVADDLLWVSMVT	1265		2022.04	
TVPRWFTTLEKA	1266		1448.78	
SVGVAPGEGPQGGPQKGTY	1267		1785.89	
EVATMDLPSFE	1268	5,Ox.[M];	1254.56	
KEGMTEEEVSRT	1269		1395.64	
VLLESDLEMLAAMKQGPG	1270		1901.98	
KSNAQMEELAAKAAL	1271		1574.83	
GVDNPGHPF	1272		939.43	
APVDVSSYELDVPA	1273		1461.70	
DFVRQVVEKL	1274		1232.70	
GPALETGSWQLPL	1275		1368.70	
REKLWHH	1276		1005.54	
MVPTQDLTPGQFVVAL	1277		1715.91	
KGAAALPEQYQDFLGK	1278		1735.91	
KEAAVKSELRLE	1279		1372.77	
GTATLYLQELWAT	1280		1466.76	
GLAVMLLGGEPL	1281		1169.66	
KGFPGWACNQKLGLN	1282		1632.84	
DPALVAHDMSLGLK	1283	9,Ox.[M];	1482.77	
KGVPNFVPLGSL	1284		1227.71	
LQLFESLGKL	1285		1147.67	
VVEVSVNPPTVLAAT	1286		1495.82	
QPPSWAFNLPNK	1287		1398.73	
DPFGQLVFRDRPFNLMTF	1288		2200.11	
KGMGSKTPDVAMGLK	1289		1519.78	
MFGEEMMFVKAE	1290		1448.62	

Sequence	SEQ ID NO	Mod. Sites	Mass	DPPIV motif
GPGAVGENGQHVN	1291		1235.57	
EVDDKLELT	1292		1061.54	
KEALGMEAWLANM	1293		1463.70	
LVDSEDLKRL	1294		1187.66	
ATCPTKKKMQM	1295	11,Ox.[M];	1282.64	
SQLDGEFKGK	1296		1108.56	
KGVLGLPEMCLYT	1297	9,Ox.[M];	1439.74	
WDDNMNKLWHH	1298	5,Ox.[M];	1511.66	
GQGPKEVHVANNAQQCYLL	1299		2069.03	
EHEPPVVWWPVL	1300		1487.75	
NPLDLDLFTMHLVDAH	1301		1850.92	
VQEVMRPLLPM	1302	5,Ox.[M];	1328.72	
QSEKLAWFFNH	1303		1406.69	
GTMDESVAKL	1304		1050.52	
PGFVGGPLGQATVGPNPT	1305		1665.87	Y
VLGDLQGEQVKF	1306		1332.72	
KSCTGLVLVSEVPMMLQQVT	1307		2163.11	
GEAGLHLDDGLVSQ	1308		1410.70	
SPCSLLTAVVDMQDM	1309		1609.72	
ELGLPQDKLEGL	1310		1311.71	
TPAAELVGMLDDGLR	1311	9,Ox.[M];	1573.80	
PAAPPEPDAAAKAL	1312		1318.71	
GPADQMVKQNAEW	1313		1473.68	
TVPHELTGPGLT	1314		1221.66	
LGAAGRGPAGTFLVLQGEMHPAPG	1315	19,Ox.[M];	2320.17	
YENELMAR	1316		1025.47	
EAGSMEQEKKLHHQ	1317	5,Ox.[M];	1667.77	
GPAAELAGTAQYLMRH	1318		1685.84	
GTAGYPSQTLPKKNNHAVPGPA	1319		2205.14	
EGGKTFPEHVKGTTVQ	1320		1714.87	
GTANGEFGRAAV	1321		1149.56	
LSKDTRSL	1322		919.53	
TELTMPELPYGT	1323		1351.65	
NVQWEAAEVQAQ	1324		1372.66	
DFSLVHRK	1325		1001.56	
VVAAPFVKRFPHVPA	1326		1634.94	
KGVDLWVL	1327		929.54	
LVWVNEEDLHTPGLAF	1328		1839.93	
PEEHPTLGMRKCHHTPL	1329		1982.98	
EPAAQGEGTVEQEKK	1330		1600.77	
LDELEDRAQELR	1331		1486.75	
SRELEELSERL	1332		1360.71	

Sequence	SEQ ID NO	Mod. Sites	Mass	DPPIV motif
SSAYLLDKLYAQ	1333		1371.72	
KMLLYDTLL	1334		1109.63	
KTTAEVELK	1335		1018.57	
TVHDDPKYMPKLL	1336		1556.81	
APGACLQADVSVVCL	1337		1445.71	
GTDDVKKAF	1338		980.51	
PGVGLLGDFHKAP	1339		1307.71	
NYLKELNYAW	1340		1313.66	
ELGHPSYVPNYTMQLM	1341	13,Ox.[M];	1895.88	
PGPMGPMSKKDGPA	1342		1369.67	
PGVEQWFEGPV	1343		1244.59	
LQWRTKYETD	1344		1339.66	
GPLGEKNESWRVLL	1345		1597.88	Y
GLDYSYVPLKHHLGY	1346		1761.89	
AFPSYTTPFAYK	1347		1392.68	
GLGAYNVDGDHDLKRT	1348		1730.86	
RFVPTPNVS	1349		1016.55	
SGPSSNLVGLK	1350		1058.58	
FHTGLQSELQGHCVL	1351		1668.83	
KGNAQEPATQLVVAL	1352		1538.86	
KVTEAPLNPK	1353		1096.64	
CYDDMEKLWHH	1354		1476.60	
LVPELDGK	1355		870.49	
EVGVFVPNYLMK	1356	11,Ox.[M];	1411.73	
EAVPPERMFDLYAQ	1357	8,Ox.[M];	1681.78	
ELSVEDHAVEGDGNELA	1358		1783.81	
KVEASGPLDRTRNM	1359	14,Ox.[M];	1589.82	Y
AAFPPDVASQADFK	1360		1463.71	
KDPLGYLLGM	1361	10,Ox.[M];	1122.59	
LLGGGMLGQNCVLALK	1362		1586.88	
GLTGFNPTGR	1363		1019.53	
KPPYDKLEDMNM	1364		1480.68	
KLDSYEEALDLH	1365		1432.69	
GPASNATLEKKMYETAL	1366		1823.92	
KEGFRLPAQK	1367		1173.67	
KGVAVMDLPMLVDKL	1368		1628.91	
KDMVDDLPSFE	1369	3,Ox.[M];	1311.58	
KAAACGLAGGAL	1370		1002.53	
KELGMPMGSLDLH	1371	5,Ox.[M];	1443.70	
ELATSREGLPAGL	1372		1313.70	
PAGVHAGPAQR	1373		1060.56	
MLDEHFVLK	1374		1131.59	

Sequence	SEQ ID NO	Mod. Sites	Mass	DPPIV motif
DLRDVLAS	1375		888.48	
GLGWWVQPLVSK	1376		1369.75	
GPAPPKFDMDLPGPA	1377		1509.74	Y
HLVHSELK	1378		962.54	
ALVWKGMPNPTVMKPL	1379	7,Ox.[M];	1797.98	
FVMSPPGELKMQ	1380		1363.68	
GLGLKNEYTLARPPT	1381		1629.91	
EELMPQVEVTK	1382	4,Ox.[M];	1318.65	
KVDYDEEEVKAK	1383		1452.72	
RLLESKMGAVK	1384		1231.73	
GQELELKLR	1385		1085.63	
GPALACALLDPQ	1386		1168.60	
KGTGTDWALGTGKTA	1387		1463.75	
GFPGLVVAGY	1388		979.52	
FALGDAETVKK	1389		1178.64	
SQKEDKYEEELKLL	1390		1751.91	
GLGGRVYDHLDEMLMK	1391		1833.88	
GLPELHLMSTPDEHLHEPVAL	1392		2335.16	
EGEEVKLW	1393		989.49	
LHVSAATDVLHMT	1394		1394.71	
AAFPPDKPQSDYKQ	1395		1591.78	
GVGGDVRHGGWHVAPG	1396		1557.78	
NSYEEALDHL	1397		1190.53	
DDMWDEKLWHH	1398		1511.65	
QELQVDDLEGSLPTTFPV	1399		1987.98	
EVPPSHWPMLTMLQ	1400		1665.80	
LPEVPECCDRKTL	1401		1502.73	
KFAVLDLLAT	1402		1090.66	
GLGMFHAVWAVLL	1403	4,Ox.[M];	1429.77	
KAEEQMRREQEAK	1404	6,Ox.[M];	1648.82	
FDLRNLLTK	1405		1119.65	
WLDKELEELSER	1406		1546.77	
LAGEFEFMCVTLGH	1407		1553.72	
GFQRLKDHVPMKHTMAP	1408	15,Ox.[M];	2009.01	
DHFLFDKPVSPL	1409		1414.74	
KKDLDDLVYFHEL	1410		1634.85	
DPPVATFPSQDYKQ	1411		1592.77	
DVMLKMLH	1412	6,Ox.[M];	1002.51	
GQVVKEHVLYAAQN	1413		1555.82	
KTTEDLLDTVAAT	1414		1377.72	
KEGWDLPAKQ	1415		1171.62	
ELGACKKKMGAM	1416		1266.65	

Sequence	SEQ ID NO	Mod. Sites	Mass	DPPIV motif
AEGVWHVGDTALHHVVGPA	1417		1951.99	
KMMPPLQSELDSVKAN	1418	3,Ox.[M];	1803.90	
EPLQVMDLGAKGPG	1419	6,Ox.[M];	1427.72	
ELAALNYGKLLPQ	1420		1429.79	
KDQSNGELLK	1421		1131.60	
PAGGESPGHSLEYMGKH	1422		1753.81	
GVFTTLEKA	1423		965.53	
ALTENWFKTPQ	1424		1334.66	
ELMRQLKFAGK	1425		1320.74	
SDCPGTLELLTGM	1426		1336.63	
LVQEVMPK	1427		943.52	
ELGGSGLVVALSV	1428		1200.67	
PGSPGPAGEAGPPAGPAWAGKA	1429		1899.94	
SPGRVTGKTEGAPALP	1430		1537.83	
GENLSQLLSK	1431		1088.60	
QELQVEDAVP	1432		1127.56	
KTELVCSSRSPLQ	1433		1447.76	
KGSVGETPMRQLGGAW	1434	9,Ox.[M];	1689.83	
GPLPGSSRGLPGPHETFGR	1435		1918.99	Y
VLLESDAAYMQALDLH	1436		1788.89	
LGGLEPPPTKGVY	1437		1327.73	
RSQPSAFDRLLGTAAM	1438		1720.89	
GTGGPSGKLWR	1439		1115.61	
QDLVQAVKAL	1440		1084.64	
EGFRLSPVVE	1441		1132.60	
EPDVLWCVTPVLVSM	1442		1687.86	
GLGFLLDGSPTGKVGGL	1443		1587.86	
KALGTGRAELK	1444		1143.68	
KTCKDVVDTVGLR	1445		1433.77	
KVLDPEPGGLASHTKVVCTGF	1446		2155.14	
DHLLNLFVLDLH	1447		1448.79	
DHPDLSPVKLRVL	1448		1488.86	
KDPLLGYVAM	1449		1106.59	
TVTNELLGPF	1450		1090.58	
GEGLEYEPWLGNPV	1451		1559.75	
EAGLMVAR	1452		846.45	
TTTTLDEAENL	1453		1207.57	
PAETSRKDVALSVEET	1454		1731.89	
ETELSDLNCALLQ	1455		1448.69	
WAYVDWEVNEKGPA	1456		1663.76	
ELKPYELTGPARQ	1457		1501.79	
ELVVDPGFDLLAAP	1458		1455.78	

Sequence	SEQ ID NO	Mod. Sites	Mass	DPPIV motif
VLSENWYLMVHYLMK	1459		1925.96	
DNEALELSRLL	1460		1272.68	
GFAGDDARMAL	1461		1123.52	
AAAAEGGVRGAAMPQ	1462		1356.67	
EPKLQNLALDGY	1463		1360.72	
DHYNLVHATLQ	1464		1310.66	
PQLGSHGGDTDFMYTLH	1465		1875.84	
EVQHRLDEAENL	1466		1452.71	
DDMEKLWHH	1467		1210.53	
EGPNVKYTLVWAE	1468		1505.76	
VDDLEMVLDANKKLWDT	1469		2004.99	
KAKTVQFDVDPPC	1470		1447.73	
VEGMLPAQK	1471		972.52	
KDAFFLDKVFR	1472		1385.75	
LVADLLR	1473		799.50	
KGVLGGELGPTVP	1474		1223.69	
PLEEVVYLVKKVVCH	1475		1754.97	
ALGEQLTVDL	1476		1058.57	
PAAESLQQFFNH	1477		1388.66	
VPLDVHLRVPSAT	1478		1403.80	
GLGACKLQTPSPL	1479		1284.70	
WVLGSWGNLLGGSL	1480		1458.78	
GFGGVLDWPPCAL	1481		1331.64	
EHLESKELCYLQSRSMMV	1482	16,Ox.[M];	2199.02	
TLADHEESKLL	1483		1255.65	
QDEWYKLSGFGL	1484		1442.70	
KAQPEAKFAGK	1485		1174.66	
ALDSLLALK	1486		943.58	
GLLLNMRKVLK	1487		1284.83	
TTFRMAGKLVPL	1488	5,Ox.[M];	1349.76	
GLVEDFKNK	1489		1049.56	
ETCFEVKAK	1490		1054.52	
KDDLLACYMP	1491		1168.54	
LSDLAHKL	1492		896.52	
DLEFRLAWSDKL	1493		1492.77	
GTADVVKLW	1494		988.55	
GPADVHFENR	1495		1141.54	
KFVLDQLASGF	1496		1224.66	
LLSRPVYESAVDPS	1497		1532.80	
GLALVYMWPDMSAPNAL	1498		1848.90	
PAGGKGPGEAPCVLF	1499		1399.71	
TFGPEAFTLMTNKDHGLLSRS	1500	10,Ox.[M];	2338.17	

Sequence	SEQ ID NO	Mod. Sites	Mass	DPPIV motif
EVAVLVPHPL	1501		1073.64	
EVLKGNELGAK	1502		1157.65	
WHLKPEKLWHH	1503		1510.79	
PHAMDACWLKKKMQ	1504	4,Ox.[M];	1702.81	
ESPGPLGPTVSELT	1505		1383.71	Y
KMGLTDVLMH	1506		1144.59	
GPAGSQYLELRQL	1507		1431.75	
LGGVFCPGKEL	1508		1119.58	
ALAVADESPSHLNKGL	1509		1621.86	
QGALPMKPAAVQ	1510	6,Ox.[M];	1226.65	
KDPLLDCPGWLLLGM	1511	15,Ox.[M];	1686.86	
KPPDYKLENMEDM	1512	10,Ox.[M];	1625.72	
PANLKKHGAVPAKGAEGGP	1513		1798.98	
TPGLNPPDDVKTAP	1514		1421.73	
KSEMYQEMLGQ	1515		1343.61	
KLACTDALQR	1516		1118.60	
LQHREVEAENL	1517		1337.68	
KFVVTDLLDEKVCVM	1518	15,Ox.[M];	1754.92	
VLLPEGQNQQGAH	1519		1390.70	
LVDPYDLGWVVALK	1520		1587.89	
KEVKEAWLLTEK	1521		1473.83	
MYPGLADMR	1522	1,Ox.[M];	1069.48	
KLLMDPMPGSL	1523		1201.63	
KTTYLKDPEHWLQ	1524		1658.86	
EFMVPVALMPYTF	1525	9,Ox.[M];	1560.76	
ENTGFEKLGLMKNMEL	1526	11,Ox.[M];	1869.91	
LGVANVDLVDKL	1527		1255.74	
DSYEEALDLH	1528		1191.51	
GFGSLWVLTMPEVQRAL	1529	10,Ox.[M];	1920.02	
VMGNLKEVTGEVAV	1530	2,Ox.[M];	1461.76	
KDLVMNAKMKGFT	1531		1482.78	
MYPGLADMR	1532	1,Ox.[M];	1069.48	
EVGLMPVDAWQTLL	1533	5,Ox.[M];	1587.81	
GLAAGRGPAGGVDPDLFTLGPTGSH	1534		2320.17	
KWTMGLPGGVNEPAP	1535		1553.79	
KDDLLACYMP	1536		1168.54	
VGALTTESELSPSH	1537		1427.71	
AHVETAYNSLM	1538		1235.58	
DHLACHNEEALDLH	1539		1616.72	
EASWGPQFLLANMK	1540		1591.80	
PVPWGTPALLMMH	1541		1449.73	
APEYAGPAQR	1542		1059.52	

Sequence	SEQ ID NO	Mod. Sites	Mass	DPPIV motif
AELRLAEATL	1543		1086.62	
TVPMMTDWVLK	1544		1320.68	
NVDVLVQPPT	1545		1081.60	
KFVLDQDASGF	1546		1226.61	
KDPGEMFLDVLYVSKVM	1547		1971.01	
AEEAEVVQEA	1548		1074.50	
EVEFFDLEVK	1549		1254.62	
PSFLEMLPTGSPSLT	1550	6,Ox.[M];	1592.78	
GPMNLFTDSEVQVPL	1551	3,Ox.[M];	1662.81	
SYEEALDLH	1552		1076.49	
VLDDTYDDPV	1553		1151.52	
KFADVNVTVLDQ	1554		1348.72	
TTTTLDEAENL	1555		1207.57	
KEVTYVLEGVKPDP	1556		1573.86	
GTPGKLHPHFNGPN	1557		1472.73	
KNPVAKMLQAFVVLESPA	1558		1942.08	
TPLTRPEEHTKPV	1559		1504.82	
PNYWFLPELK	1560		1306.68	
LQETLDNMWFLKLCH	1561		1890.91	
GLPTWKLLYKPL	1562		1428.86	
KVDLKMYQVEAENL	1563		1679.87	
VEFPGVPSLH	1564		1081.57	
VPGTLQGLTMPR	1565		1269.69	
KFAALVPDFDWPTPSPF	1566		1934.98	
EVASNDAKKKLAQR	1567		1557.87	
EGLVGQAQVDKQLFHLP	1568		1879.01	
RVAPEEHPVN	1569		1147.59	
KLGQCLVDASERVGL	1570		1587.86	
VEAAAVELHVGLVF	1571		1453.80	
GLGRAESMYLF	1572		1243.61	
NEAAAPHHHSPPG	1573		1321.61	
LVSTLEETLERMELGAK	1574	12,Ox.[M];	1935.01	
PGPVVPSLKEPGP	1575		1273.72	
DPTEYPLSPDRMKVT	1576		1748.87	
WDDMMEKLWHH	1577		1527.65	
EVSLEVLYK	1578		1079.60	
GPGKWATKAVSLPVSK	1579		1625.95	
KTELACKWALTPA	1580		1431.76	
DVESLYVPNAL	1581		1219.62	
GPGLNVMPVSNTT	1582	7,Ox.[M];	1302.65	
GTSVFTTLEKGTTH	1583		1478.75	

Sequence	SEQ ID NO	Mod. Sites	Mass	DPPIV motif
PASALMMVDTVRAL	1584	6,Ox.[M];7, Ox.[M];	1506.77	
GPTGLTRSDLPGTLH	1585		1521.80	
GVHTSEAGSKGP	1586		1126.56	
PGLRMLPNLLAL	1587	5,Ox.[M];	1323.79	
SFGPGTLDPVSF	1588		1223.60	
ALDMETVVALLRAAVY	1589		1734.95	
GPTNPLTGPGFSHF	1590		1428.68	
KSLWRLQLEAK	1591		1371.80	
EVACLLEEAKKKLRSPK	1592		1942.11	
KGYLLALQTLLETQ	1593		1590.91	
TVRHHFTTLDAK	1594		1425.77	
STYDTKAAGAP	1595		1081.52	
ELGSVTTLDTVR	1596		1290.69	
SPEGHPDLANFE	1597		1312.59	
KELCQVGTVDGF	1598		1295.63	
KHGTEPLVMELM	1599		1384.70	
LLGDVQSLGVSL	1600		1200.68	
EGLLMNKNTPSEQVEPA	1601		1856.92	
KPPDDSQPYCVYVSGPMLVYQ	1602		2386.12	
KDDLLNDQPL	1603		1170.61	
LAGEFERWYPGET	1604		1554.72	
ATGGPTVMQKKESQTLH	1605		1812.94	
VEGRGPTTAAEVD	1606		1301.64	
TVEKLPAACTLGH	1607		1339.71	
KDTSEKFTGLAP	1608		1293.67	
KLVWYRTHNVEDMKALDL	1609		2231.18	
GQVTMQDPLSMKDVLRGLG	1610	5,Ox.[M];	2061.05	
EVAGQESLNKTL	1611		1288.67	
GEGAGLWMLFYN	1612	8,Ox.[M];	1373.61	
ATLQDRVL	1613		915.53	
SEAAAMDMRRSHAPV	1614	8,Ox.[M];	1644.76	
GPLADGSLEKVHALGPME	1615	17,Ox.[M];	1836.93	Y
QVAKDADFSHKTKQVKL	1616		1943.08	
VPVALWELYDENMTPT	1617	13,Ox.[M];	1893.89	
TVPHELTGPGKS	1618		1222.65	
KPDPLDAPLNPK	1619		1304.73	
QAGLAMFHGPL	1620		1141.58	Y
KLFALQGDLVNAAP	1621		1456.81	
EHNLGELVNELLAACV	1622		1723.86	
FGLMENNKFMVQ	1623		1457.69	
EVGRLPVVDPEGAQQHTLAPG	1624		2170.12	

Sequence	SEQ ID NO	Mod. Sites	Mass	DPPIV motif
PEEHLGPTLL	1625		1105.59	
NVLGEPLDERGWVLK	1626		1724.92	
KAPYDLLLADEAKQ	1627		1574.83	
GPVDALVMVLPGVDAL	1628		1565.86	
GLDLNVDLQYRLGH	1629		1612.84	
GDAVNKLVEDSW	1630		1332.64	
EFATLGPHTMGL	1631		1273.63	
KACRFPMQDALQKKMK	1632		1923.01	
KGMHELANKM	1633	3,Ox.[M];	1174.58	
EGDKKAAVEDGKGF	1634		1450.72	
DPEVKPVLELK	1635		1266.73	
KGGDEELKNVTN	1636		1303.64	
GMDERLLPMMGLW	1637	9,Ox.[M];	1564.73	
EPLVNMKMKMQ	1638	6,Ox.[M];	1364.68	
TAGGDTTHTVEQEKK	1639		1601.78	
LAGDELLAANPL	1640		1196.65	
SMFEWWGLPK	1641		1280.62	
KGAQEESTWAAHK	1642		1442.70	
SVGVPGPQPEFT	1643		1214.61	
PAASVQSTNKF	1644		1149.59	
GPSSVQTNFMYPGLTSADV	1645		1970.91	
VPFTFVVTTLGSN	1646		1381.73	
PVDAADTVRSL	1647		1143.61	
DEALKDVPSGN	1648		1144.54	
LLGAFFCVPDHNDVDLK	1649		1902.94	
GFTNDEKLGL	1650		1093.56	
LALLNMCALDLH	1651	6,Ox.[M];	1342.68	
PAGDHVAAVPL	1652		1046.56	
KAGYMWLLPAGP	1653		1303.68	
KPTTVLLGDPV	1654		1139.66	
STELFK	1655		724.39	
KGCCAYEEVKAR	1656		1356.65	
KSAGFLTHFPAHTLGMASK	1657	16,Ox.[M];	2017.05	
LREDLELR	1658		1043.58	
GLCKKMEKLWHH	1659		1509.79	
GQDLMLENR	1660		1075.52	
GLGVGNAVEVVNCVQ	1661		1457.73	
GLRPEEHPTLLT	1662		1362.74	
GPLQEQNGPEGEELVH	1663		1732.81	Y
YVGPTYLEDMVALNDTVLTPL	1664	10,Ox.[M];	2340.16	
KAGYAGLLTTYSTVKAK	1665		1772.00	
KGVLGLGCLVK	1666		1086.67	

Sequence	SEQ ID NO	Mod. Sites	Mass	DPPIV motif
KLEPMVRDLKEKL	1667		1598.92	
TLGMTLETLGTM	1668		1267.63	
LLTNWDDPYQLQKTGM	1669	16,Ox.[M];	1938.92	
TVSAGQADEAYLK	1670		1352.67	
GPPMFMADLRPEPY	1671		1620.74	
KDTVYLLK	1672		979.58	
KGGPCLVLDPPKKGFQ	1673		1683.94	
KEGKLGMDLAWTRNSTLVAQQ	1674		2346.23	
